# A pyroptosis proportion tunable nano-modulator for cancer immunotherapy

**DOI:** 10.7150/thno.112209

**Published:** 2025-07-25

**Authors:** Zhuang Chen, Zuo Yang, Zhiping Rao, Yi Luo, Weijing Liu, Chaoqiang Qiao, Qian Jia, Peng Yang, Ruili Zhang, Zhongliang Wang

**Affiliations:** Lab of Molecular Imaging and Translational Medicine (MITM), Engineering Research Center of Molecular & Neuro-imaging, Ministry of Education, School of Life Science and Technology, Xidian University, Xi'an, Shaanxi, 710126, P. R. China.

**Keywords:** pyroptosis, apoptosis, nanomedicine, photothermal therapy

## Abstract

**Rationale:** Pyroptosis, a form of programmed cell death mediated by gasdermin proteins, holds significant potential in cancer immunotherapy. However, precise control of pyroptosis in cancer cells is essential to avoid biosafety concerns. This study aimed to develop a tumor-targeted and tunable pyroptosis-inducing strategy to enhance antitumor efficacy while minimizing systemic side effects.

**Methods:** An innovative H_2_S-activated nanomodulator equipped with an optical switch was designed for tumor-specific and adjustable pyroptosis induction. The nanomodulator was activated by H_2_S in the tumor microenvironment of colorectal cancer and further regulated by laser irradiation. Gasdermin-E-mediated pyroptosis was triggered through the synergistic effects of photothermal temperature modulation and demethylation. The proportion of cells undergoing pyroptosis was precisely controlled within a tunable range.

**Results:** The nanomodulator successfully induced pyroptosis in microsatellite-stable colorectal cancer cells within a tunable range of 0-31%. This precise regulation significantly enhanced antitumor efficacy while minimizing systemic side effects. The combination of photothermal modulation and demethylation ensured effective and safe pyroptosis induction.

**Conclusions:** This study presents a novel and precise method for controlling pyroptosis using photothermal temperature modulation. The findings provide essential guidance for in vivo applications and offer valuable insights into the development of nanomedicines capable of safely and effectively inducing adjustable proportion of pyroptosis in cancer therapy.

## Introduction

Recent advances in emerging pyroptosis-mediated therapy (PMT) have demonstrated great potential for the immunotherapy of low-immunogenicity tumors such as triple-negative breast cancer, pancreatic cancer, and microsatellite-stable (MSS) colorectal cancer (CRC) 1-3. Although this approach offers a unique advantage in antitumor immunity, accumulating evidence shows that pyroptosis can also be a central contributor to various inflammatory diseases, including pathogenic infections, atherosclerosis, and multiple organ failure 4-8. Therefore, PMT may harm normal tissues and organs, leading to undesirable inflammatory side effects. Current research primarily focuses on limiting the location of pyroptosis at spatial and temporal scales to minimize side effects 9. However, it is crucial to recognize that pyroptosis not only triggers local inflammation, but also leads to the release of IL-1β and IL-18, which recruit additional inflammatory cells and amplify the overall inflammatory response. For instance, during the COVID-19 pandemic, although pyroptosis was predominantly observed in the lungs, its excessive occurrence and subsequent release of inflammatory cytokines can lead to cytokine storms, multiple organ failure, or even fatality 10,11. It can be inferred that in addition to restricting the location of pyroptosis, adjusting the proportion of cells undergoing pyroptosis to an optimal range is equally important for enhancing the safety of PMT. However, the optimal range of pyroptosis in cancer therapy remains to be clearly defined. Additionally, there is a lack of research focused on the precise regulation of the proportion of pyroptosis.

Crosstalk between apoptosis and pyroptosis has been reported in recent studies 12-14. Traditional apoptotic triggers or chemotherapeutic drugs might be endowed with the ability to induce pyroptosis because gasdermin E (GSDME) can be specifically cleaved by activated caspase-3 and switch the route of cell death to pyroptosis in both human and murine cells. GSDME is silenced in most cancer cells owing to promoter methylation, which limits the conversion of apoptosis to pyroptosis 15. The combined use of demethylating agents can upregulate GSDME expression, thereby reactivating the conversion 16. In this pathway, caspase-3 activation is a critical factor that acts as a molecular switch from apoptosis to pyroptosis. Therefore, precise spatiotemporal control of caspase-3 activation provides a feasible way to adjust the proportion of GSDME-induced pyroptosis. Photothermal therapy (PTT) is a valuable platform owing to its precise control capabilities 17-19. By finely tuning the PTT temperature, it is possible to adjust the activation of caspase-3 and further control the proportion of GSDME-induced pyroptosis. Therefore, there is an urgent need to develop a pyroptosis proportionally tunable nanomodulator to precisely regulate the proportion of pyroptosis.

**Scheme 1:**
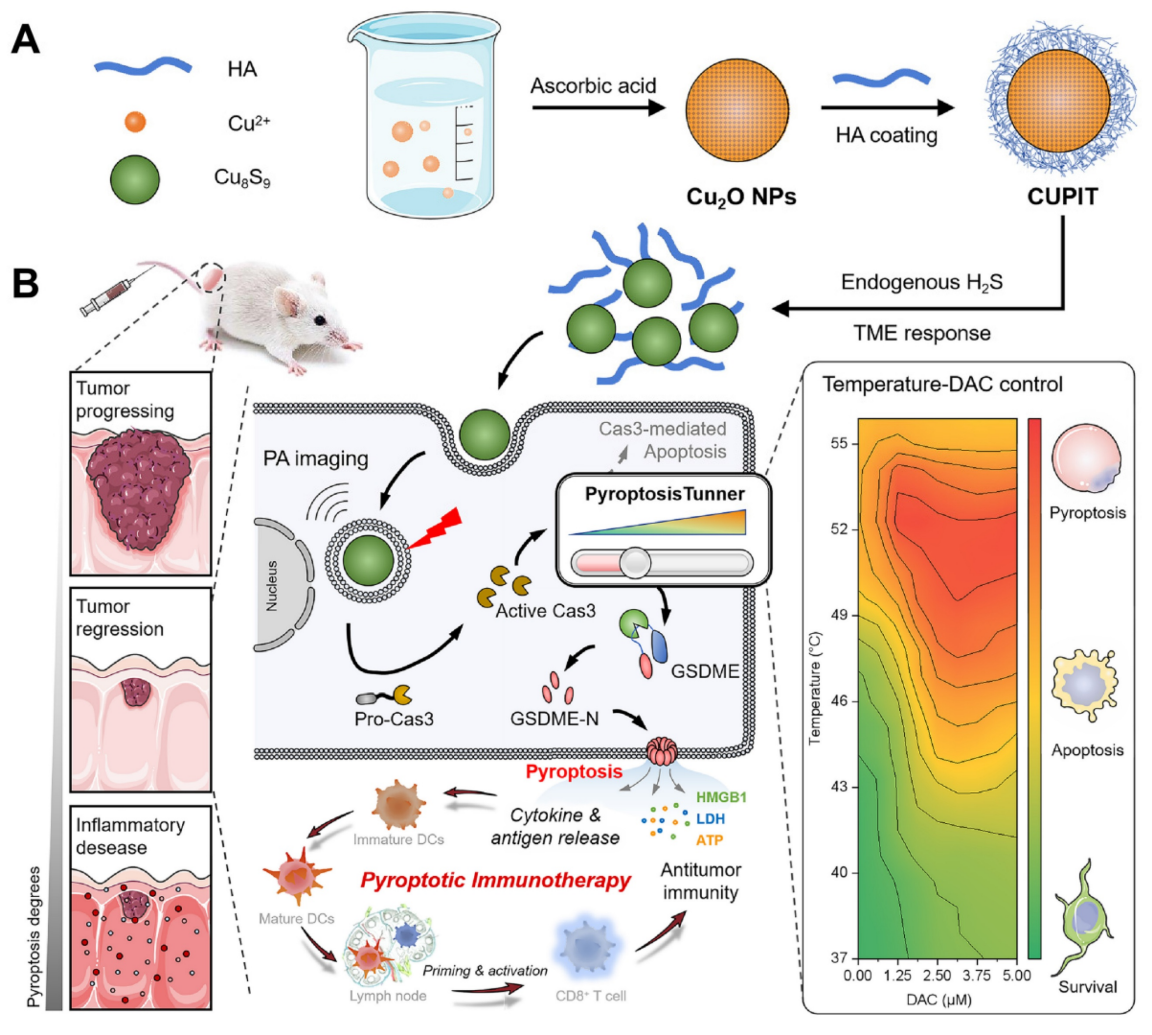
** Schematic illustration of CUPIT-mediated tunable pyroptosis for antitumor immunotherapy.** (A) Synthesis of CUPIT NPs. (B) CUPIT NPs are intravenously injected and accumulated in tumors. In the hydrogen sulfide-rich microenvironment of colorectal cancer, CUPIT nanoparticles transform in situ into Cu_8_S_9_, exhibiting desirable PTT properties. The relationship between varying temperatures and the proportion of pyroptosis was investigated by adjusting the heating temperature, providing guidance for subsequent therapies. Copper-based pyroptosis-immunotherapy trigger, CUPIT; nanoparticles, NPs; photothermal therapy, PTT.

In this study, we developed a pyroptosis proportion-tunable nanomodulator in a mouse model of MSS CRC to meet the need for location and proportion dual-controllable pyroptosis (Scheme 1). Briefly, cuprous oxide nanoparticles were selected as a PTT precursor and coated with hyaluronic acid (HA) to form a copper-based pyroptosis-immunotherapy trigger (CUPIT). CUPIT nanoparticles (NPs) exhibited minimal absorbance in the near-infrared (NIR-II) region. However, once CUPIT NPs accumulate at the CRC tumor site, they are converted into Cu_8_S_9_ NPs by endogenous H_2_S in the CRC tumor microenvironment (TME) and show a strong absorption peak in the NIR-II region. This H_2_S activated photothermal property ensures spatial and temporal specificity of the TME 20. The upregulation of GSDME expression via decitabine (DAC) ensures the necessary protein context for PMT. By precisely controlling the PTT temperature, we achieved fine regulation of the proportion of cells undergoing pyroptosis via the GSDME-driven pathway. Using this CUPIT platform, the relationship between PTT temperatures and percentage points of pyroptosis (PPP) was quantitatively defined, achieving a tunable range of 0-31%. By analyzing the pathological characteristics and therapeutic effects of pyroptosis in mice, we found that pyroptosis induced at a proportion of 18.8% effectively triggered anti-tumor immune responses while minimizing the risk of systemic inflammation. In murine CRC models, this CUPIT-based approach led to subcutaneous tumor eradication, prolonged survival, and significant inhibition of distant metastatic CRC when combined with an anti-PD1 antibody (αPD-1). This platform offers a robust and measurable reference for the precise implementation of PMT, balancing efficacy and safety in cancer therapy.

## Methods

*Materials:* Copper chloride dihydrate (CuCl_2_•2H_2_O) and sodium ascorbate were purchased from Sinopharm Chemical Reagent Co., Ltd.; Hyaluronic acid sodium salt (HA) and polyvinyl pyrrolidone (PVP) were purchased from Beijing Innochem Technology Co., Ltd.; Decitabine (DAC) was purchased from Shanghai Bide Medical Technology Co., Ltd.; The cell culture medium and fetal bovine serum were purchased from Gibco Life Technologies; The western-blot antibodies such as Anti-DFNA5/GSDME, Anti-Cleaved Caspase-3 and β-Actin were purchased from Abcam Trading Co. Ltd. The flow cytometry antibodies such as APC Anti-Mouse CD8a, PE/Cyanine7 anti-mouse CD4 and FITC anti-mouse/human CD44 were purchased from Biolegend; The test kits and enzyme-linked immunization kits were purchased from Shanghai Biyuntian Biotechnology Co., Ltd.; All the cell lines were purchased from Shanghai Zhong Qiao Xin Zhou Biotechnology Co., Ltd.; All mice were purchased from the Animal Center of the Fourth Military Medical University (Xi'an, China). In this study, animal protocols were reviewed and approved by the Fourth Military Medical University's Institutional Animal Care and Use Committee (approval number: 20220284).

*Preparation of CUPIT*: CuCl_2_•2H_2_O (17.5 mg) and PVP (1 g) were dispersed in 16 mL of deionized water and vigorously stirred for 30 min under magnetic stirring to obtain solution A. Subsequently, sodium ascorbate (16.3 mg) was dissolved in 4 mL of deionized water and sonicated to obtain solution B. Then, solution B was slowly added to solution A under ultrasound conditions (65 W, 5 min). After mixing fully, the reaction mixture was sonicated for another 25 min (55 W). The products were collected by centrifugation (12000 rpm, 10 min), washed three times with deionized water, and then redispersed with 10 mL of deionized water to obtain Cu_2_O. Then, 10 mg HA was added to the above solution and stirred overnight using a magnetic stirrer. The products were collected by centrifugation and named CUPIT.

*Sulfidation process of the CUPIT in vitro:* NaSH·xH_2_O was used to simulate endogenous H_2_S. The CUPIT (150 μg mL^-1^) was added to NaSH·xH_2_O (200 μg mL^-1^) solution at room temperature under shaking for 30 min. Then, the products were collected by centrifugation (12000 rpm, 10 min). The obtained nanoparticles were characterized by XRD and TEM.

*Vis-NIR absorption of CUPIT in the presence of NaSH·xH_2_O:* The Vis-NIR absorption of CUPIT in the presence of NaSH·xH_2_O was studied by changing the concentrations of NaSH·xH_2_O. Aqueous CUPIT (100 μg mL^-1^) solution was added into NaSH·xH_2_O solution of various concentrations (0-1 mM) at room temperature under shaking for 30 min. Then, the Vis-NIR absorption of the mixtures was measured.

*Photothermal effect of CUPIT in the presence of NaSH·xH_2_O in vitro:* The mixtures were prepared by mixing CUPIT solution (100 μg mL^-1^) with NaSH·xH_2_O (0-1 mM) and shaking at room temperature for 30 min. Then, 1 mL of CUPIT (100 μg mL^-1^) and the resulting mixtures were irradiated with a 1064 nm laser (1 W cm^-2^). Meanwhile, an infrared camera was used to record the heating curves.

*Photoacoustic image of CUPIT in the presence of NaSH·xH_2_O:* The mixtures were prepared by mixing CUPIT solution (100 μg mL^-1^) with NaSH·xH_2_O (0-1 mM) and shaking at room temperature for 30 min. Then, photoacoustic images of CUPIT (100 μg mL^-1^) and the above solutions were acquired using a real-time whole-body mouse imaging scanner, MSOT In Vision 256-TF (iThera-Medical GmbH, Munich, Germany).

*Cell culture:* A mouse colon carcinoma cell line (CT26.WT) was cultured in RPMI-1640 medium containing 1% penicillin-streptomycin double antibody and 10% fetal bovine serum in a 37 °C, 5% CO_2_ incubator. All the above cellular manipulations were performed at a sterile ultra-clean table.

*MTT Assay:* CT26.WT cells were seeded into a 96-well plate and cultured for 12 h. The cells were then divided into six groups: PBS (G1), DAC (G2), Cu_8_S_9_ (G3), DAC+Cu_8_S_9_ (G4), Cu_8_S_9_+Laser (G5), and DAC+Cu_8_S_9_+Laser (G6). After 24 h of incubation, 10 μL of 3-(4,5-dimethylthiazol-2-yl)-2,5-diphenyltetrazolium bromide (MTT) solution was added to each well. Following 4 h of incubation, the medium was removed, and 150 μL of dimethyl sulfoxide was added, and the absorbance was measured using a microplate reader. The concentrations of DAC and Cu_8_S_9_ were 2.5 μM and 50 μg mL^-1^, respectively. Additionally, the laser power density was set at 1 W/cm^2^ with an irradiation time of 8 min.

*Western blot assay:* The expression of pyroptosis-related proteins (GSDME-FL, GSDME-N, and c-Cas3) in CT26.WT cells, with or without DAC treatment, was analyzed by western blotting. After treatment, cells were washed and lysed to extract total protein. Equal amounts of protein samples were separated by SDS-PAGE and transferred onto PVDF membranes. The membranes were then blocked in PBS containing 5% skim milk for 1 hour, followed by incubation with the respective primary antibodies for 1 hour. After washing, the membranes were incubated with the appropriate fluorescently labeled secondary antibodies for another hour. Finally, the expression levels of pyroptosis-related proteins under different conditions were analyzed, with β-actin used as the loading control.

*ELISA analysis:* The pro-inflammatory molecule IL-1β in the cell culture medium suspension of differently treated groups was detected by ELISA. The differently treated cells were centrifuged at 100 g for 5 min to obtain the supernatants. Then, 100 µL of each sample and standard at different concentrations were added to 96-well plates and incubated for 2 h. After washing the plate, 100 µL of biotinylated antibody was added to each well and incubated for 1 h at room temperature. Next, horseradish peroxidase-labeled streptavidin was incubated in the wells for 30 min at room temperature in the dark. Finally, 50 µL of stop solution was added to each well, and the absorbance was read at a wavelength of 450 nm within 15 min. IL-1β, IL-6, and TNF-α in serum samples were determined using a similar method.

*LDH release assay:* The amounts of LDH in the cell culture medium were evaluated using an LDH Release Assay Kit (Beyondtime) according to the manufacturer's instructions. CT26.WT cells were seeded into a 96-well plate and cultured for 12 h. The cells were then divided into six groups: PBS (G1), DAC (G2), Cu_8_S_9_ (G3), DAC+Cu_8_S_9_ (G4), Cu_8_S_9_+Laser (G5), and DAC+Cu_8_S_9_+Laser (G6). After 24 h of incubation, the supernatant was collected and centrifuged at 400g for 5 min at 4 °C, and stored on ice. Subsequently, 120 µL of the supernatant was transferred to a new 96-well plate and mixed with 60 µL of LDH detection solution. After incubating in the dark for 30 min, the absorbance was measured at 490 nm.

*ATP release assay:* The amounts of ATP in the cell culture medium were evaluated using an ATP Assay Kit (Beyondtime) according to the manufacturer's instructions. CT26.WT cells were seeded into a 96-well plate and cultured for 12 h. The cells were then divided into six groups: PBS (G1), DAC (G2), Cu_8_S_9_ (G3), DAC+Cu_8_S_9_ (G4), Cu_8_S_9_+Laser (G5), and DAC+Cu_8_S_9_+Laser (G6). Supernatants were obtained by centrifugation at 400g for 5 min. A new 96-well plate was prepared, and 100 µL of ATP detection reagent was added to each well and incubated for 5 min at room temperature (25 °C). Then, 20 µL of the supernatant from each sample was added to the corresponding wells and mixed quickly. The luminescence intensity of each group was measured using a microplate reader, and ATP release fold changes were calculated using the PBS group as the reference.

*In vitro Annexin V-FITC/PI assay:* To evaluate the death mechanism of different samples, the Annexin V-FITC/PI assay was carried out using the Annexin V-FITC Apoptosis Detection Kit (Beyondtime). CT-26.WT cells were seeded into 6-well plates and incubated for 12 h. The cells were then divided into seven groups: PBS (G1), DAC (G2), Cu_8_S_9_ (G3), DAC+Cu_8_S_9_ (G4), Cu_8_S_9_+Laser (G5), and DAC+Cu_8_S_9_+Laser (G6). After incubation for another 24 h, the cells were stained with Annexin V-FITC/PI according to the instruction of the kit and then analyzed through FACS. Fluorescence emission of Annexin V-FITC and PI were were detected by FL1 (Ex: 488 nm, Em: 500-540 nm) and FL2 (Ex: 488 nm, Em: 570-680 nm) channels, respectively.

*Morphological study of pyroptosis after different temperature treatment:* The cell morphology of differently treated groups was observed using an inverted fluorescence microscope. CT-26.WT cells were seeded into a 96-well plate and incubated for 12 h. Then the cells were co-incubated with or without DAC in fresh media for 24 h, The cells in each group were irradiated with laser to maintain 37 °C, 43 °C, 46 °C, 49 °C, 52 °C and 55 °C for 8 min, respectively. After incubation for another 12 h, the cell morphology was observed. The pyroptotic cell death showed swelling and bubble-like protrusions. Apoptotic cells were recognized by cell shrinkage, nuclear chromatin condensation. At least 200 cells were counted for each sample, and a percentage of pyroptosis cells was calculated as the ratio of pyroptotic cell death to total cell count.

*H&E staining:* After different treatments (DAC + 37 °C, DAC + 43 °C, DAC + 46 °C, DAC + 49 °C), mice were sacrificed on the third day. Organs were removed and fixed with 10% formalin, embedded in paraffin, sectioned at 5 μm and stained with hematoxylin and eosin for pathology. The samples were imaged by a microscope.

*Animal body temperature measurement:* The animal body temperatures were monitored by measuring on the tail root with a handheld digital thermometer.

*Survival study:* Twenty CT-26 tumor mice were randomly divided into four groups. DAC was administered by intraperitoneal injection, 15 μg per mouse for 3 consecutive days, each group was irradiated with laser to maintain 37 °C, 43 °C, 46 °C and 49 °C for 8 min, respectively. The animals were checked every 12 h, and a total study period was 72 h.

*In vivo anti-tumor therapy:* All animal experiments were carried out according to guidelines for laboratory animals established by the Xidian University Center for Animal Experiment. Tumor model: the female Balb/c mice (4-5 weeks old) were subcutaneously injected with CT-26.WT cells (5 × 10^6^ per mouse). When tumors reached an average volume of 100 mm^3^, mice were randomly divided into six groups (*n* = 5). Then the tumor-bearing mice were treated with PBS (G1), DAC (G2), CUPIT (G3), DAC + CUPIT (G4), CUPIT + Laser (G5) and DAC + CUPIT + Laser (G6), respectively (dosage: DAC, 15 μg per mouse for 3 days; CUPIT, 200 μg per mouse; irradiated with laser to maintain 46 °C for 8 min). The tumor volume was defined as: V = ((length) × (width)^2^)/2. After another 2 weeks of therapy, residual mice were sacrificed. The tumor tissues and the main organs were collected and stored at -80 °C for further examination.

*Anti-tumor immune response analysis on CT-26.WT tumor-bearing mice:* On the fifth day of treatment, the mice were sacrificed. Fresh tumors, spleens, and draining lymph node tissues were collected for immunocyte analysis via FACS. Briefly, the tumor tissues were digested in 2 mL of RPMI 1640 medium containing 2% FBS, 1 mg mL^-1^ collagenase IV, 0.1 mg mL^-1^ hyaluronidase, and 0.1 mg mL^-1^ DNase I for 3 h at 37 °C. The resulting suspension was filtered through a 70 μm nylon cell strainer. Red blood cells (RBCs) were then removed using a Red Blood Cell Lysis Buffer, and the cells were washed with PBS. Relevant antibodies were added, and the mixture was incubated at 4 °C for 30 min with gentle shaking. Prior to FACS detection, the cells were filtered through a 300-mesh metallic strainer.

*Distal antitumor effect in vivo:* BALB/c mice received subcutaneous injection of 5 × 10^6^ CT-26.WT cells at the right flank (the primary tumor) and 5 × 10^5^ cells at the left flank (distant tumor) to build bilateral CT-26.WT tumor-bearing mouse model. 7 days post tumor inoculation, mice were randomly divided into 4 groups (6 mice per group) of PBS, αPD-1, pyroptosis and αPD-1 + pyroptosis. After that, primary/distant tumor size and mouse weight were recorded for 21 days after treatment, and mouse survival was investigated 60 days after tumor inoculation. Simultaneously, a bilateral CT-26.WT tumor-bearing mouse model was built and mice received the same treatments 7 days after tumor inoculation. 48 h after treatments, distant tumors and spleens in each group were collected for analysis.

*Statistical Analysis:* Statistical analyses were performed using GraphPad Prism software. Data are presented as mean ± standard deviation (SD), with error bars indicating SD. The mean and SD values were calculated from three or more independent experiments. Comparison between the two groups was made by Student's two-tailed t-test, and the comparison between the two groups of curves was conducted using multiple independent samples t-tests. Statistical significance was established as indicated *P < 0.05; **P < 0.01; ***P < 0.001 and ****P < 0.0001.

## Results and Discussion

### Synthesis and characterization of CUPIT

**Figure 1:**
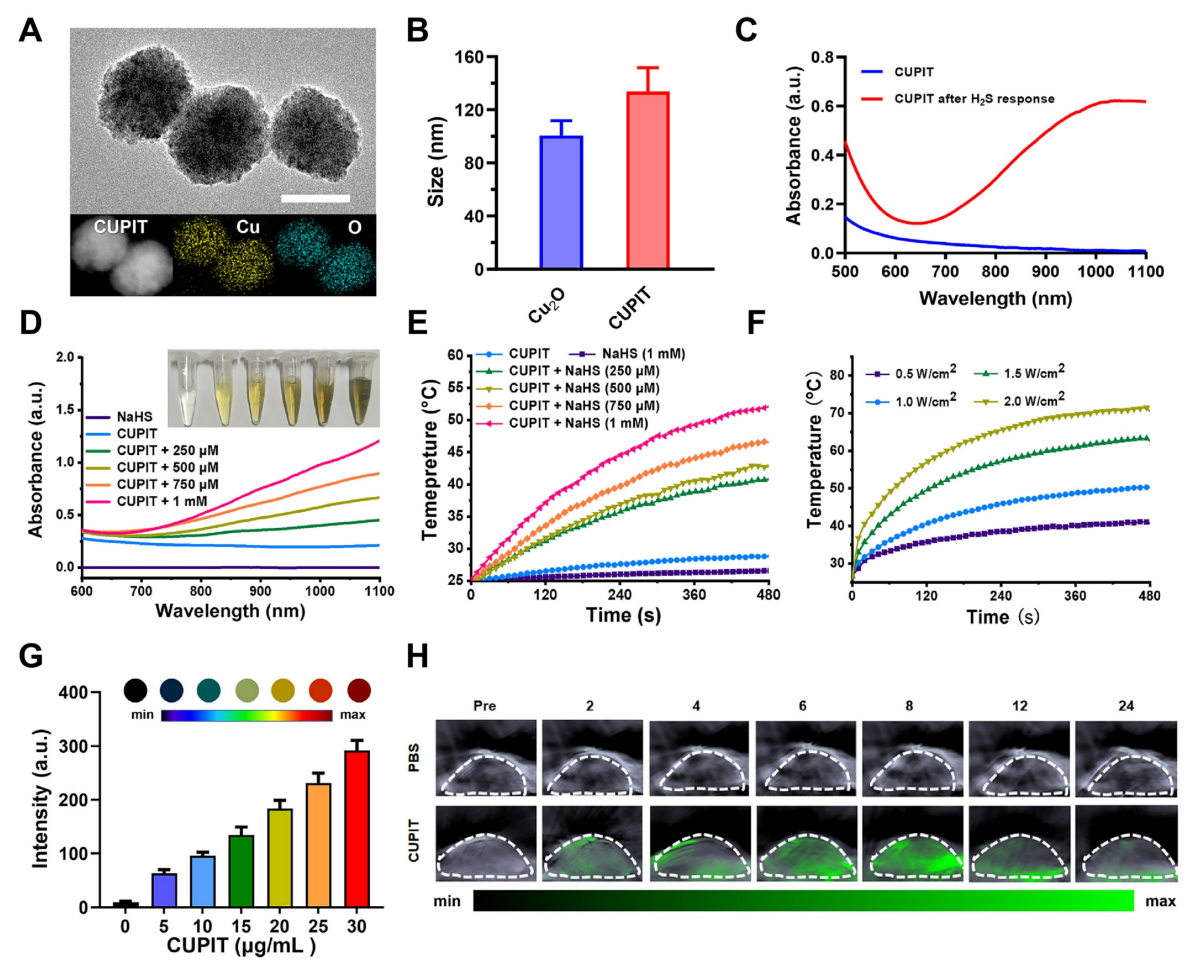
** Synthesis and characterization of CUPIT.** (A) Transmission electron microscopy image and elemental mapping of CUPIT NPs; scale bar: 100 nm. (B) Hydrodynamic diameter of CUPIT NPs. (C) The absorption spectra of CUPIT (blue line) and CUPIT + NaHS (red line). (D) The absorption spectra of CUPIT in different concentrations of NaHS solution. (E) PTT heating curves of CUPIT in different concentrations of NaHS solution. (F) PTT heating curves of CUPIT under different laser power density. (G) PA signal intensities of different concentrations of CUPIT solution in the presence of 1mM of NaHS. (H) PA imaging at different time points (pre: pretreatment; time points range from 2 to 24h) after injection of CUPIT. Copper-based pyroptosis-immunotherapy trigger, CUPIT; nanoparticles, NPs; photoacoustic, PA; photothermal therapy, PTT.

First, we synthesized the core composition of CUPIT, in which cuprous oxide (Cu_2_O) is the PTT pro-drug. Then, the Cu₂O nanoparticles were coated with HA, resulting in a negative surface charge (Figure S1, Supporting Information) and enhanced specific binding affinity for CD44, which is overexpressed on CT26.WT cells 21,22. The prepared CUPIT NPs were spherical with a homogeneous diameter of approximately 120 nm (Figure 1A-B), which was suitable for a better enhanced permeability and retention (EPR) effect 23-25. The valence state of Cu in CUPIT was confirmed to be Cu(I) (Figure S2, Supporting Information). No significant aggregation or size changes of CUPIT were observed after incubation in PBS, culture medium, or medium supplemented with serum for 48 h, demonstrating its good stability for prolonged blood circulation (Figure S3, Supporting Information). Meanwhile, hemolysis test results further demonstrated that CUPIT caused negligible hemolysis and exhibited good blood compatibility (Figure S4, Supporting Information). Subsequently, the H_2_S responsiveness of CUPIT was investigated, showing an approximately 74.4-fold absorbance enhancement in the NIR-II region (1064 nm) (Figure 1C), whereas other typical reducing substances present in organisms led to a bare change in optical absorbance (Figure S5, Supporting Information). The substance generated after the response was confirmed to be Cu_8_S_9_ (Figure S6, Supporting Information). This conversion to Cu_8_S_9_ results in a strong enhancement of near-infrared absorption, which is mainly attributed to the localized surface plasmon resonance (LSPR) properties of Cu_8_S_9_, whose intensity is much higher than that of CUPIT 26, 27. Surprisingly, the size sharply decreased to < 10 nm in response to NaHS, indicating the potential of Cu_8_S_9_ for deep tumor penetration. We believe that this phenomenon results from a multi-step mechanism involving the initial formation of hollow structures, followed by particle downsizing, in which both the Kirkendall diffusion effect 28 and the dissolution-reprecipitation process 29 play key roles (Figure S7, Supporting Information). To ensure the controllability of the heat-driven switch, the controllable response to H_2_S was measured. As shown in Figure 1D, increased optical absorbance was positively correlated with increased NaHS concentration (mimicking H_2_S in the TME), and this increase was linearly correlated within the NaHS concentration range of 0-1000 μM 30 (Figure S8, Supporting Information).

Subsequently, under 1064 nm laser irradiation, the photothermic effect of Cu_8_S_9_ was highly dependent on both concentration (0-100 μg mL^-1^) and laser power density (0.5-2.0 W cm^-2^). Furthermore, laser irradiation on-off cycle test validated the superior PTT stability of Cu_8_S_9_ (Figures 1E-F and Figure S9, Supporting Information). The maximum heating temperature of CUPIT reached 52 °C with a NaHS concentration of 1000 μM, ensuring the induction of apoptosis in tumor cells. Moreover, absorption in the NIR region facilitated the generation of a photoacoustic (PA) signal 31, which was positively correlated with the generated Cu_8_S_9_ concentration (Figure 1G and Figure S10, Supporting Information). This provided a more accurate spatial area selection for tumor-specific PTT guided by *in vivo* multispectral optoacoustic tomography (MSOT) imaging (Figure 1H).

### Necessary conditions for CUPIT to initiate apoptosis-pyroptosis switching in CT26.WT cells

**Figure 2:**
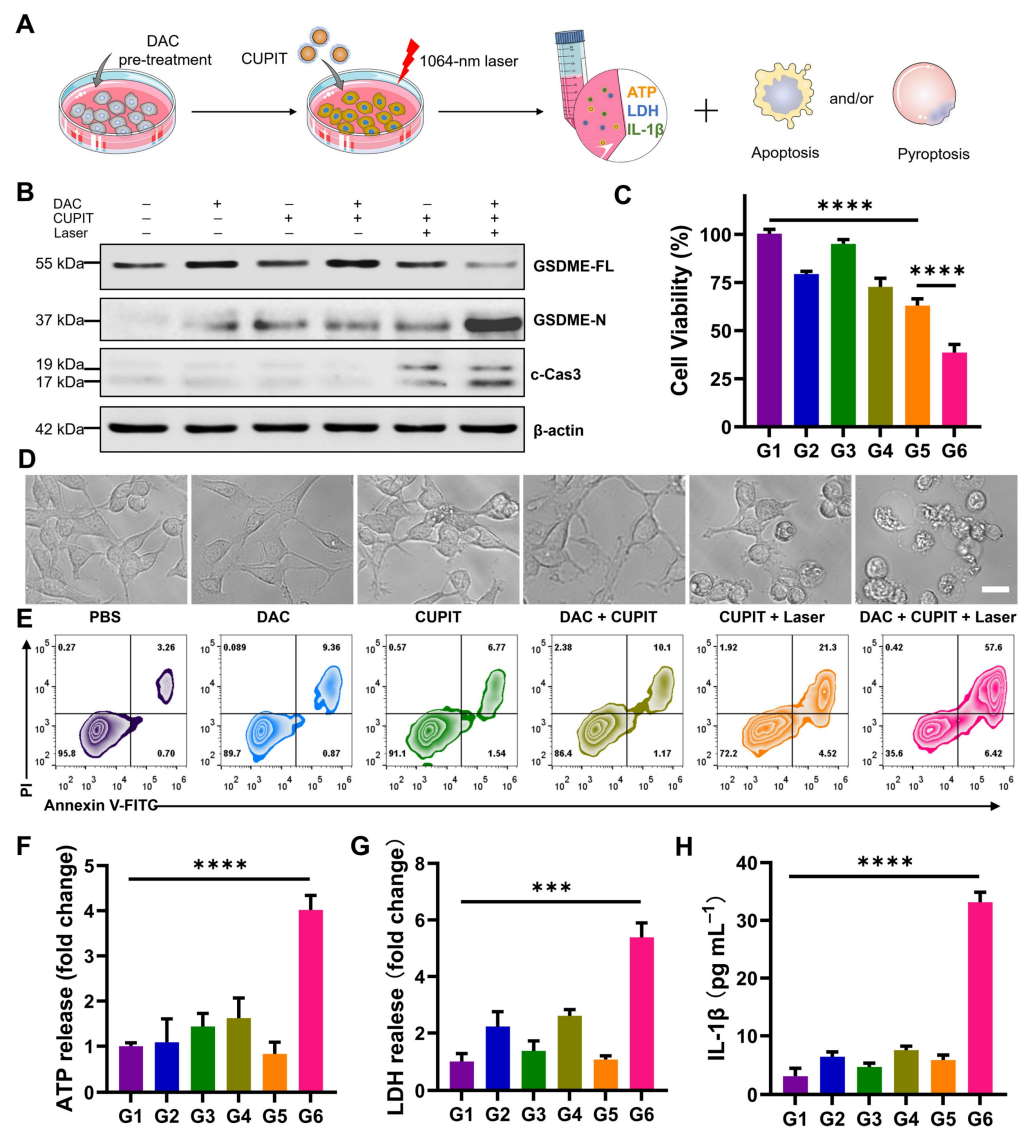
** Necessary condition for CUPIT to initiate the apoptosis-pyroptosis switch in CT26.WT cells.** (A) Schematic illustration of the apoptosis-pyroptosis switch mediated by CUPIT. (B) Western blotting of GSDME-FL, GSDME-N and c-Cas3 expression in CT-26.WT cells after different treatments. (C) The cytotoxicity assessment on CT-26.WT cells after different treatments (*n* = 5). (D) Representative photographs of CT-26.WT cells after different treatments; scale bar: 25 μm. (E) Flow cytometry analysis of propidium iodide (PI) and Annexin V- FITC-stained CT26.WT cells. The release of (F) ATP, (G) LDH, and (H) IL-1β after different treatments (*n* = 3). Data are presented as the mean ± SD. Significance was calculated via unpaired *t*-test (***P < 0.001; ****P < 0.0001). Copper-based pyroptosis-immunotherapy trigger, CUPIT; gasdermin E, GSDME; full-length GSDME, GSDME-FL; GSDME N-terminus, GSDME-N; cleaved caspase-3, c-Cas3.

To examine whether the prepared CUPIT could induce pyroptosis, the PTT killing effect of CT26.WT cells was evaluated *in vitro* (Figure 2A). Since CT26.WT cells express little GSDME due to the methylation of its promoter, the DNA demethylation reagent DAC was used beforehand to induce demethylation of the promoter region in CT26.WT cells. Consistent with previous reports 32, 2.5 μM DAC was selected for pre-treatment, and CCK8 assays confirmed that this concentration only caused a slight inhibition of cell growth (Figure S11, Supporting Information). The groups were as follows: G1 group: PBS; G2 group: DAC; G3 group: CUPIT; G4 group: DAC + CUPIT; G5 group: CUPIT + laser; and G6 group: DAC + CUPIT + laser. To understand the mechanism of CUPIT-mediated pyroptosis, the expression of full-length GSDME (GSDME-FL), GSDME N-terminus (GSDME-N), and cleaved caspase-3 (c-Cas3) in CT26.WT cells after different treatments was evaluated by western blotting (Figure 2B). The expression of GSDME was upregulated after 3 days of pre-treatment with 2.5 μM DAC, but decreased following further treatment with CUPIT under laser irradiation. Although CUPIT treatment under laser irradiation upregulated the expression of c-Cas3 in CT26.WT cells, it did not result in significant GSDME cleavage compared to the group treated with DAC and CUPIT under laser irradiation. This indicated that DAC pretreatment is necessary for inducing pyroptosis in CT26.WT cells, and that c-Cas3 upregulation induced by PTT is crucial in driving this process. In addition, the MTT results showed a consistent range of decreased cell viability and upregulated c-Cas3 expression in the different groups, especially in G5 and G6 (Figure 2C), further emphasizing the pivotal role of c-Cas3 in mediating cell death mechanisms.

To directly assess pyroptosis in CT26.WT cells transitioning from the caspase-3-mediated apoptotic pathway, representative cell imaging was conducted. This method allowed us to observe the characteristic features of pyroptosis, including evident cell swelling and emergence of bubbles from the plasma membrane. Neither DAC nor CUPIT treatment alone significantly affected cell morphology, whereas CUPIT treatment combined with laser irradiation induced morphological changes in apoptotic cells. CT26.WT cells underwent pyroptosis only when combined with DAC pretreatment, CUPIT, and laser irradiation (Figure 2D). To further confirm the photothermal-induced pyroptosis, cells were stained with Annexin V/PI to assess cell cytotoxicity (Figure 2E), while ATP, IL-1β, IL-18 and lactate dehydrogenase (LDH) release analyses (Figures 2F-H and Figure S12, Supporting Information) were performed as hallmarks of pyroptosis 33-35. All evidence pointed to the implementation of the G6 group triggering the strongest pyroptosis. The importance of DAC lies in its ability to effectively alter the epigenetics of CT26.WT cells, upregulating GSDME expression and providing the foundation for the occurrence of pyroptosis. We further investigated the forms of cell death induced by the photothermal effect mediated by CUPIT in GSDME-low expressing normal HUVEC cells and GSDME-high expressing normal HUASMC cells. The results showed that apoptosis occurred in HUVEC cells, whereas pyroptosis was observed in HUASMC cells (Figure S13, Supporting Information). In addition, to exclude the potential effects of laser treatment on cell death, we also investigated two additional groups: (1) a laser only group and (2) a DAC + laser group (Figure S14, Supporting Information). The results showed that these two groups exhibited no significant differences in cell morphology or cytotoxicity compared to the control group. These results further indicated that the up-regulation of GSDME expression by DAC pretreatment, coupled with its subsequent activation via the PTT effect, is a necessary condition for the switch from apoptosis to pyroptosis.

### PTT temperature regulation of CUPIT-mediated pyroptotic switch

**Figure 3:**
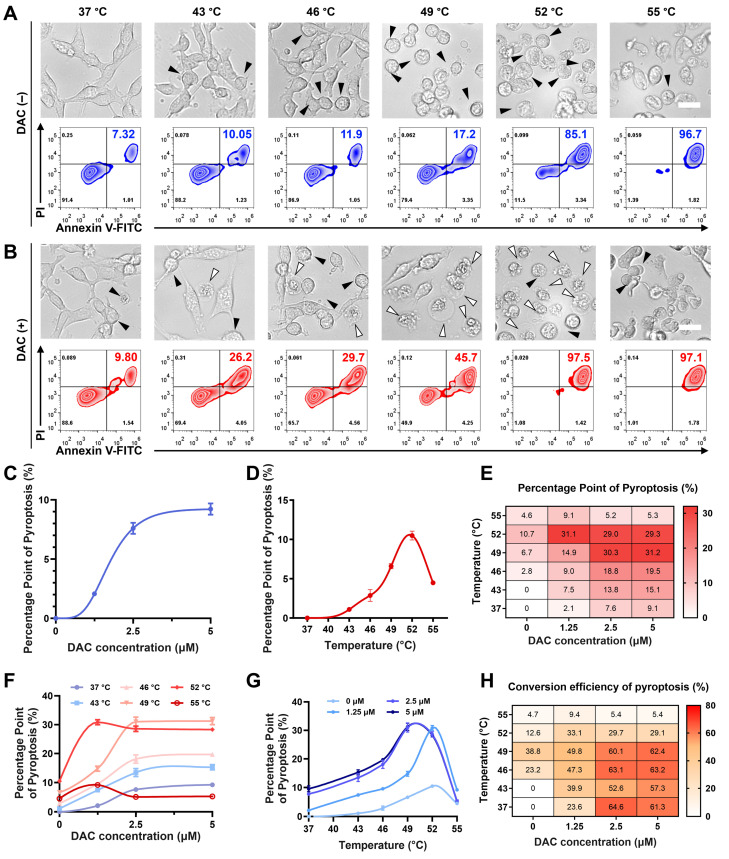
** PTT temperature regulation of the CUPIT-mediated pyroptotic switch.** (A) Representative photographs and flow cytometry analysis of CT26.WT cells after treatment with various temperatures without DAC pre-incubation, the white arrows point to pyroptotic cells, and black arrows point to apoptotic cells; scale bar: 25 μm. (B) Representative photographs and flow cytometry analysis of CT26.WT cells after treatment with various temperatures with DAC pre-incubation, the white arrows point to pyroptotic cells, and black arrows point to apoptotic cells; scale bar: 25 μm. (C) PPP *vs* DAC concentrations curve. (D) PPP *vs* heating temperatures curve. (E) Heatmap of PPP combined with different heating temperatures and DAC concentrations. (F) Effect of DAC concentrations on PPP in CT26.WT cells at different heating temperatures. (G) Effect of heating temperatures on PPP in CT26.WT cells pre-treated with different DAC concentrations. (H) Heatmap of CRP combined with different heating temperatures and DAC concentrations. Copper-based pyroptosis-immunotherapy trigger, CUPIT; conversion rate of pyroptosis, CRP; decitabine, DAC; percentage points of pyroptosis, PPP.

Studies have shown that DAC can increase intracellular GSDME expression, potentially leading to pyroptosis via cell senescence and apoptosis 36,37. Therefore, it is important to first exclude the possibility that DAC pre-treatment and PTT influence each other before investigating their crosstalk in the induction of pyroptosis. The PTT potency of CUPIT was investigated at distinct temperatures without DAC pre-treatment to ensure that temperature served as an independent variable. As shown in Figure 3A, representative cell images revealed an increase in apoptosis with rising temperatures, as indicated by the formation of apoptotic bodies, while little pyroptosis was observed.

In contrast, although cells after DAC pre-treatment exhibited little pyroptosis without laser irradiation, the number of pyroptotic cells clearly increased as the temperature increased from 43 °C to 52 °C, but disappeared at 55 °C (Figure 3B). To further elucidate the temperature-dependent cell death mechanisms in the DAC-pretreated group, we conducted Western blot analyses for c-Caspase3, GSDME-FL, and GSDME-N. The results revealed that, upon DAC pretreatment, the levels of c-Caspase3 and GSDME-N gradually increased, whereas GSDME-FL decreased as the temperature rose. Notably, the levels of GSDME-N at 49 °C, 52 °C, and 55 °C were comparable, without significant difference among these higher temperature groups. This suggests that once a certain threshold temperature was reached, further increases did not markedly enhance GSDME cleavage at the protein level. However, despite the similar protein cleavage at these temperatures, the morphological features of pyroptosis diminished at 52 °C and disappeared at 55 °C. To distinguish between pyroptosis and necrosis under these conditions, we further measured LDH and IL-1β release in the DAC-pretreated cells. LDH release, an indicator of general membrane disruption, increased consistently with temperature, while IL-1β release, a hallmark of pyroptosis, peaked at 49 °C and then declined at higher temperatures. These results suggest that although the protein markers of pyroptosis remain at high levels, excessively high temperatures shift the dominant cell death mode from pyroptosis to rapid necrosis, which may limit the complete execution of pyroptosis (Figure S15, Supporting Information). Additionally, the effect of DAC pretreatment on temperature-mediated cell death was characterized by flow cytometry (Figure S16, Supporting Information). DAC pretreatment triggered a partial increase in cell-killing, suggesting that pyroptosis may be a more efficient mechanism than apoptosis for killing cancer cells. MTT assays yielded comparable results (Figure S17, Supporting Information). Together, these results suggest that DAC pretreatment is necessary as it provides the GSDME protein, and there is a non-monotonic relationship between pyroptosis and temperature, which is more complex than that of photothermal-mediated apoptosis.

PPP was defined as the ratio of pyroptotic cells to the total cell count within a unit area of view. The conversion rate of pyroptosis (CRP) was defined as the ratio of pyroptotic cells to the total dying cell count to describe the efficiency of the apoptosis-pyroptosis switch. We further examined distinct concentrations of DAC and PTT temperatures in detail to understand their effects on PPP and CRP. For DAC pre-treatment, the PPP vs. DAC concentration curve exhibited an "S-like shape" (Figure 3C) and showed a concentration plateau (PPP approaching 9.1%) beyond 2.5 μM, indicating decreasing marginal utility and suggesting an optimal concentration range of ≤ 2.5 μM. In contrast, the PPP vs. temperatures curve displayed a monotonically increasing trend from 37 to 52 °C (Figure 3D), indicating a similarly significant but more sensitive and adjustable influence of temperature on PPP compared to DAC. To obtain a more systematic relationship, heat maps of PPP, total cell death (TCD), and percentage points of apoptosis (PPA) were generated (Figure 3E and Figures S18 and S19, Supporting Information). First, the effects of DAC concentration and PTT temperature on PPP were systematically analyzed using these heat maps. The combined effects of DAC and PTT resulted in nonlinear changes in PPP, which were limited independently by each variable, and the maximum was approached under conditions of 49 °C and DAC ≥ 2.5 μM (Figures 3F-G). Furthermore, PPA was only influenced by temperature, with no significant changes observed at different DAC concentrations, further indicating that the effect of DAC on apoptosis was negligible.

To further elucidate the CUPIT-mediated apoptosis-pyroptosis switch, a heat map of CRP was established based on its definition (Figure S20, Supporting Information). Intriguingly, different patterns of the CRP vs. temperatures curve emerged at low DAC concentrations (≤ 1.25 μM) and at high DAC concentrations (≥ 2.5 μM), although DAC itself benefited CRP in a manner similar to PPP. At low DAC concentrations, CRP increased gradually with temperature, peaking at 49 °C, and then decreased. In contrast, at high DAC concentrations, CRP showed little change below 46 °C and also decreased after 49 °C. Moreover, the CRP under high DAC pretreatment conditions were generally higher than those at lower concentrations. This underscores the significant contribution of DAC to the CUPIT-mediated apoptosis-pyroptosis switch by acting as the provider of GSDME. The reduction of CRP above 49 °C suggested that excessive temperature imposes a limiting effect on the switch, which highlights the central role of PTT temperature as a “Ballast-like” operon module in the pyroptotic process. The obtained data were further analyzed to identify the optimal temperature range for precise regulation of pyroptosis. At temperatures below 43 °C, very limited apoptosis and pyroptosis were observed, regardless of DAC pretreatment concentration. This phenomenon is attributed to the poor activation of c-Cas3 under low-temperature conditions, which indicates that the apoptosis-pyroptosis switch requires a certain threshold of c-Cas3. Although CRP values from 37 °C to 49 °C approached a maximum at a DAC concentration of 2.5 μM, PPP values below 7.6% at 37 °C were insufficient for antitumor therapy. Interestingly, both PPP and CRP demonstrated a monotonic increase within the temperature range of 43 °C to 49 °C, providing a clearer regulatory range for constructing the desired CUPIT-mediated apoptosis-pyroptosis switch.

### Pathological characteristic changes in mice triggered by different proportions of pyroptosis in tumors

Pyroptosis within tissues and organs, including tumor tissues, induces immune activation and inflammatory reactions ranging from chronic inflammation to cytokine storms 38,39. Such responses, which are linked to the levels of inflammatory cytokines released by pyroptotic tissues, underscore the importance of controlling the proportion of pyroptotic cells at the tumor site. This suggests that the demand for tumor-targeted pyroptosis induction is no longer limited to location-dependent cell death, but also requires controllability of the proportions of pyroptosis, which is crucial for biosafety.

**Figure 4:**
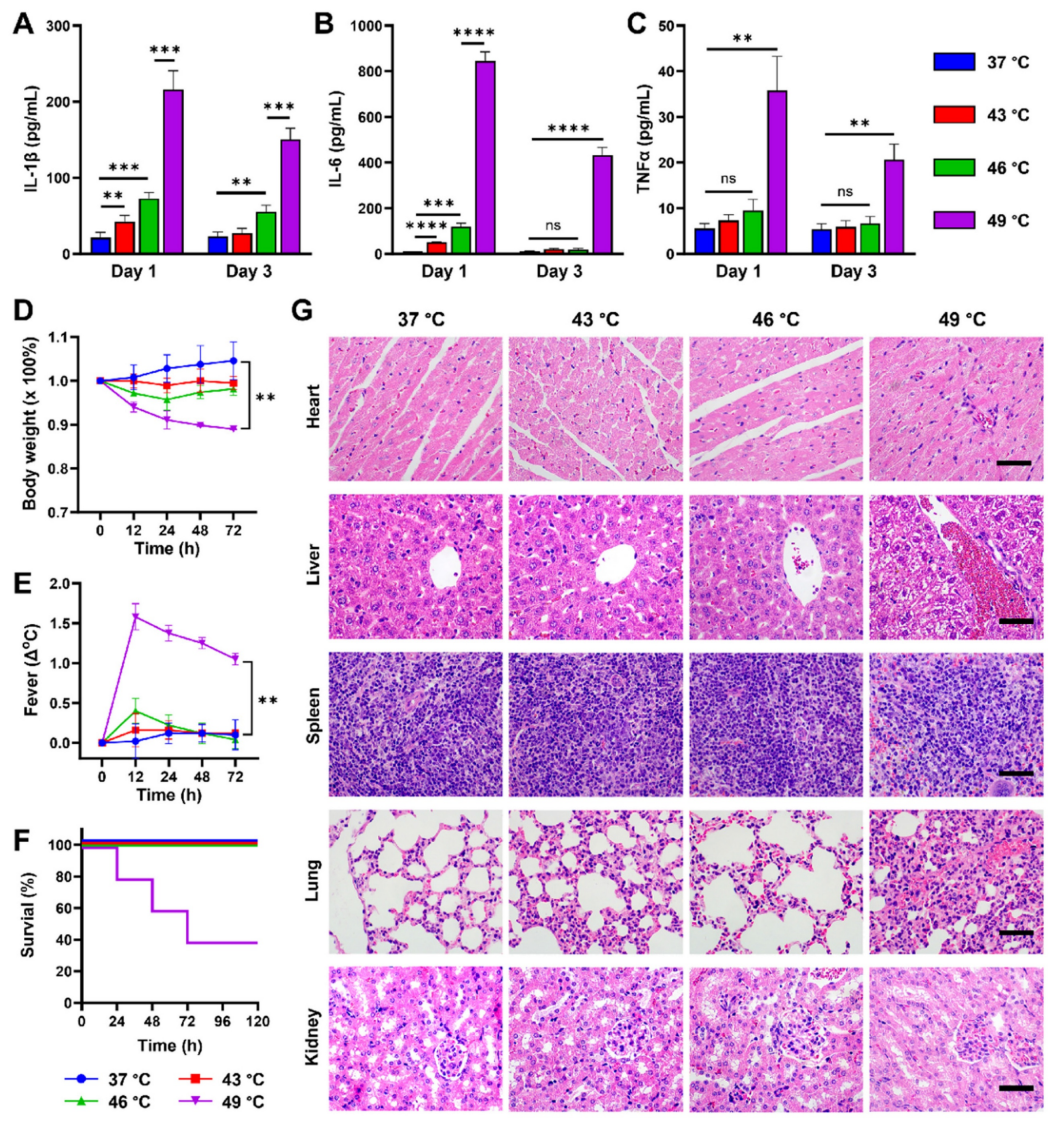
**Pathological characteristic changes of mice triggered by different proportions of pyroptosis in tumors.** After pyroptosis activation by different temperatures, serum levels of (A) IL-1β, (B) IL-6, and (C) TNF-α were measured by ELISA (n = 5). (D) Weight loss and (E) body temperature changes of mice after pyroptosis activation by different temperatures (n = 5). (F) Survival curves of the mice after pyroptosis activation by different temperatures (n = 5). (G) Pathological changes in the major organs of mice after pyroptosis activation at different temperatures, scale bar: 50 µm. Data are presented as the mean ± SD. Statistical significance was calculated via unpaired t-test and multiple independent samples t-tests. (ns, not significant; **P < 0.01; ***P < 0.001 and ****P < 0.0001).

To verify whether the established PPP value *in vitro* could be used as a reference for quantifying pyroptotic proportions *in vivo*, CUPIT-induced pyroptosis was measured in CT26.WT tumor-bearing mice. The mice were administered 200 μg kg^-1^ DAC intravenously daily for three days and then divided into four groups, each subjected to different PTT temperatures. Assuming that varying proportions of pyroptosis were triggered by CUPIT in tumor-bearing mice under different PTT temperature conditions at 37 °C (as control), 43 °C, 46 °C, and 49 °C, we precisely maintained the tumor temperature at the designated levels by tuning the laser power density during irradiation. As an example, we provided detailed thermal imaging and heating curve data at 46 °C, which clearly demonstrate that accurate and real-time temperature control at the tumor site can be achieved in vivo by adjusting the laser power density throughout the photothermal treatment (Figure S21, Supporting Information). Serum IL-1β, IL-6, and TNF-α levels were then determined by enzyme-linked immunosorbent assay (ELISA) to characterize the systemic inflammatory response induced by pyroptosis (Figure 4A-C). The results showed a temperature-dependent gradient increase in serum levels of IL-1β, IL-6, and TNF-α at 24 h post-irradiation by laser. These findings are consistent with the mapping relationship between temperature and PPP, as depicted in Figure 3G. Additionally, serum levels of IL-1β, IL-6, and TNF-α in the 49 °C group exhibited a sharp rise compared to the other groups, leading to a severe systemic inflammatory response characterized by fever and weight loss (Figure 4D-E). Notably, these elevated serum levels did not return to normal even after 72 h, with sustained fever and weight loss, resulting in 60% mortality within 3 days (Figure 4F). To further clarify the origins of these elevated inflammatory cytokines, immunohistochemistry (IHC) analysis was performed to assess the expression of IL-1β and IL-6 in tumor tissues from both the PTT (CUPIT alone) group and the pyroptosis (CUPIT combined with DAC pre-treatment) group at 46 °C and 49 °C (Figure S22, Supporting Information). It was found that the expression levels of both IL-1β and IL-6 in tumor tissues were significantly higher in the pyroptosis group compared to the PTT group, with a particularly dramatic increase in IL-1β observed in the 49 °C pyroptosis group. However, the increase in IL-6 expression within the tumor tissue was relatively modest. These findings suggest that massive pyroptosis primarily leads to the release of IL-1β within the tumor, while the elevated serum IL-6 is more likely produced by macrophages or monocytes activated as part of the systemic inflammatory response.

Given that multiple organ failure is commonly associated with severe inflammatory processes 40,41, we performed a histopathological analysis of mice from different groups 72 h after PTT-mediated pyroptosis induction. Hematoxylin and eosin (H&E)-stained sections exhibited significant inflammatory cell infiltration into the myocardial tissue, pulmonary alveoli, and around the portal vein, showing typical multiple organ injuries such as hepatocyte ballooning, degeneration in the liver, loss of delineation between the red and white pulp, alveolar septum widening in the lung, and tubular epithelial cell hyperplasia in the kidney (Figure 4G). These results provide experimental evidence that variations in PTT are correlated with the proportion of pyroptosis, highlighting the instructive role of the PPP. Furthermore, the potential risk of a systemic inflammatory response underscores the need for controlling the pyroptotic cell proportion to ensure the safety of PMT. Notably, these risks were caused by the pyroptosis of tumor cells rather than the PTT itself (Figures S23-S25, Supporting Information). Instead of posing a safety risk, a temperature of 46 °C achieved a balance of sufficient pyroptosis with tolerable side effects, making it the chosen temperature for subsequent *in vivo* application.

### Anti-tumor effect of CUPIT-mediated therapy *in vivo*

Considering the precise tunability of CUPIT-mediated pyroptosis, we used this nanomodulator for *in vivo* cancer therapy. PA imaging was used to determine *in vivo* CUPIT-H_2_S reaction kinetics using a CT26.WT murine model. Irradiation at 8 h post-injection yielded the strongest PA signal, indicating a rapid phase of tumor enrichment and the H_2_S response of CUPIT within this time frame, which led to a sharply enhanced absorbance and was beneficial in terms of PTT effect (Figure 1H). Further tumor temperature monitoring during PTT demonstrated a rapid phase of photothermal conversion of CUPIT within 5 min (Figure S26, Supporting Information). To further explore the behavior of CUPIT in vivo, we also investigated the pharmacokinetics and systemic distribution of CUPIT (Figure S27, Supporting Information). The plasma half-life of CUPIT was measured to be approximately 3.98 h. ICP-MS analysis showed a rapid increase in copper concentration within tumor tissue over the first 8 h after injection, with the tumor-to-normal tissue copper ratio reaching 7.77 at 8 h, indicative of strong tumor-targeting ability. Copper levels in major metabolic organs such as the liver, spleen, and kidney decreased significantly from the first to the second day after injection, suggesting efficient systemic clearance. By day 7, copper levels in organs were comparable to baseline values, indicating nearly complete metabolism of CUPIT. Collectively, these results demonstrate that CUPIT possesses excellent in vivo targeting efficiency, favorable pharmacokinetics, and effective metabolic clearance, providing a solid foundation for its application in cancer therapy.

**Figure 5:**
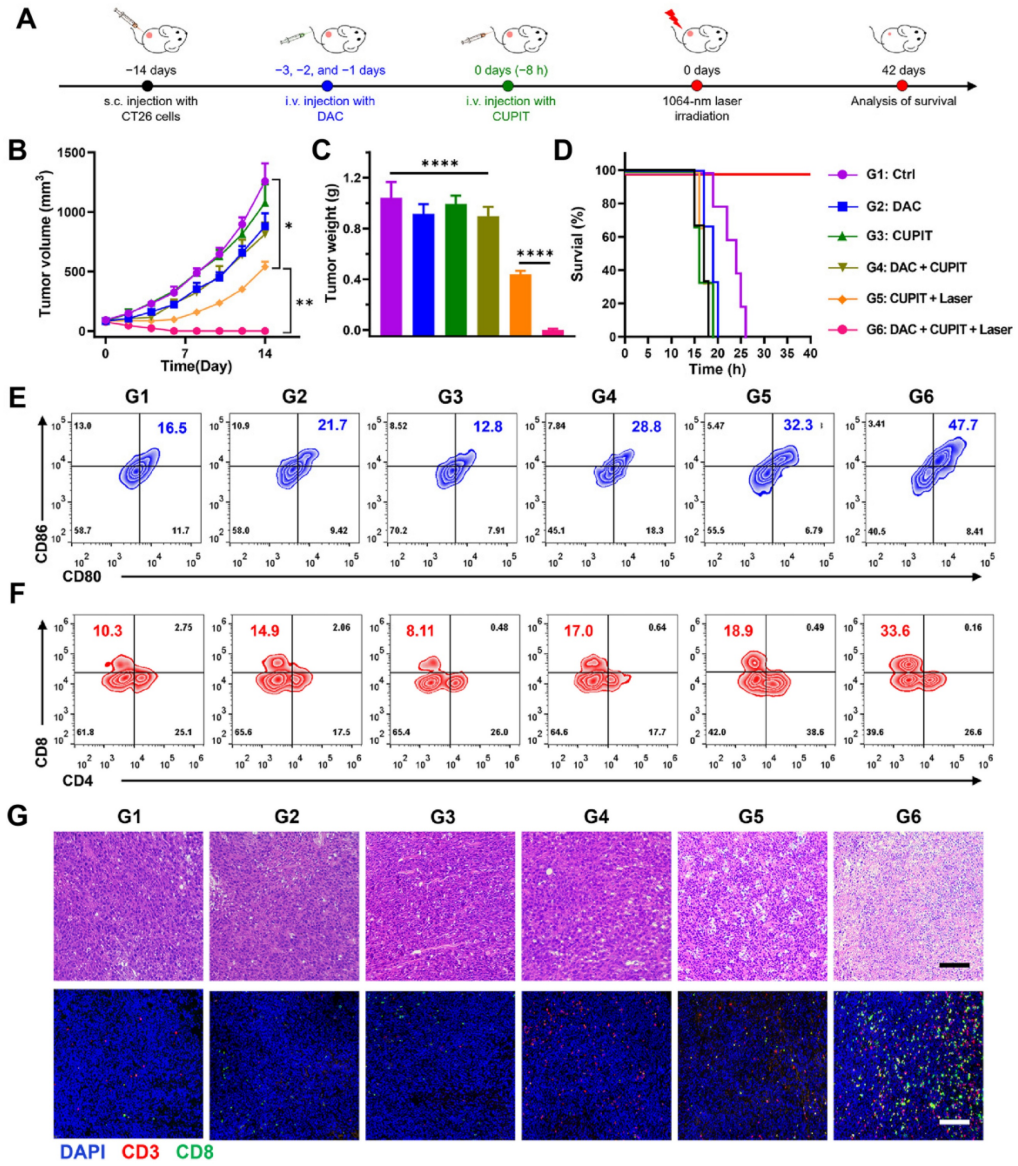
** Anti-tumor effect of CUPIT-mediated therapy *in vivo*.** (A) Schematic illustration of CUPIT-mediated therapy schedules. (B) Tumor growth curves after different treatments (*n* = 5). (C) Tumor weights at the end of experiment. (D) Survival rate of mice receiving different treatments (*n* = 5). (E) Representative flow cytometric analysis showing the percentages of matured DCs (CD11c^+^, CD80^+^, and CD86^+^) in the TTDLN (*n* = 3). (F) Representative flow cytometric analysis showing the percentages of CTLs (CD3^+^ and CD8^+^) in the tumor tissues (*n* = 3). (G) H&E staining and immunofluorescence of CTLs in the tumor tissues after different treatments; scale bar: 200 µm. Data are presented as the mean ± SD. Statistical significance was calculated via multiple independent samples t-tests and unpaired *t*-test. (*P < 0.05, ** P < 0.01, and ****P < 0.0001). Copper-based pyroptosis-immunotherapy trigger, CUPIT; cytotoxic T lymphocytes, CTLs; dendritic cells, DCs; draining lymph node, DNL; hematoxylin and eosin, H&E.

The anti-tumor efficacy of CUPIT was evaluated in a CT26.WT subcutaneous tumor model (Figure 5A). Tumor-bearing mice were randomly divided into six groups once the tumors reached approximately 100 mm^3^: the DAC group (G2), CUPIT group (G3), DAC plus CUPIT group (G4), CUPIT with laser irradiation group (G5), DAC plus CUPIT with laser irradiation group (G6), and blank control (G1). The results revealed that the G6 group showed complete tumor elimination, with 100% tumor-free mice at the end of the study. Although the G5 group (equal to PTT therapy) exhibited significant inhibition of tumor growth compared to the blank control, DAC, CUPIT, and DAC plus CUPIT without irradiation groups, there were no tumor-free mice or survival prolongation longer than one week (Figure 5B-D). To verify the role of the immune system in CUPIT-mediated therapy, flow cytometry was used to analyze the immune cells in tumor tissues and tumor draining lymph nodes (TDLN). Flow cytometry results showed that the proportion of mature dendritic cells (mDCs; CD11c^+^, CD80^+^, and CD86^+^) in the TDLN was highest in the G6 group (47.7%, as shown in Figure 5E), indicating enhanced antigen presentation in this group. The increase in mature dendritic cells likely facilitated the activation and recruitment of cytotoxic T lymphocytes (CTLs; CD3^+^ and CD8^+^). Further analysis confirmed that the proportion of CTLs in the G6 group reached 33.6%, which was significantly higher than the 10.3% observed in the blank control group (Figure 5F). Previous studies 42,43 have established the pivotal role of CTLs in anti-tumor immune responses. These findings collectively suggest that the induction of pyroptosis not only promotes more efficient antigen presentation, but also markedly enhances CTL infiltration and anti-tumor immune activity at the tumor site. In addition, immunofluorescence staining was performed to evaluate the infiltration of mature dendritic cells in tumor tissues. The results showed that mature DCs were significantly more abundant in both the G5 and G6 groups compared to the other groups (Figure S28, Supporting Information), further supporting the enhanced antigen presentation and local immune activation mediated by immunogenic cell death and pyroptosis within the tumor microenvironment.

Although immune activation was also observed in the G5 group, immunofluorescent staining revealed significantly fewer infiltrating T lymphocytes in the G5 group than in the G6 group (Figure 5G), which explained the poor efficacy in the G5 group. Based on these results, we conclude that CUPIT-mediated therapy is highly relevant for subcutaneous tumor treatment efficacy and immune activation within the tumor region. To better understand how different treatments affect the tumor immune environment, we examined the levels of PD-L1, IFN-γ, and Granzyme B in tumor samples from each group (G1-G6) by IHC (Figure S29, Supporting Information). PD-L1 expression was similar across all groups, suggesting that PTT and the induction of pyroptosis did not significantly change PD-L1 levels in tumors. However, IFN-γ was noticeably higher in all treated groups compared to the control, indicating that the presence of DAC or CUPIT in tumors helped boost local immune activity. Notably, Granzyme B, a protein released by activated cytotoxic T cells, was much higher in the G5 group and increased even further, with a broader distribution, in the G6 group. This suggests that triggering pyroptosis leads to a much stronger anti-tumor immune response, which may explain the better treatment outcomes observed with this approach.

In addition to its significant role in anti-tumor outcomes, CUPIT-mediated therapy has demonstrated additional benefits. The absence of body weight loss and inflammatory lesions involving major organs suggested that the pyroptotic switch induced by CUPIT at 46 °C not only achieved effective tumor elimination, but also minimized systemic toxicity (Figures 4D-E and Figure S30, Supporting Information). In addition, blood routine and biochemical data further confirmed the safety of CUPIT-mediated pyroptosis therapy (Figure S31, Supporting Information).

### CUPIT-mediated pyroptosis for potentiating immune checkpoint inhibitor therapy

**Figure 6:**
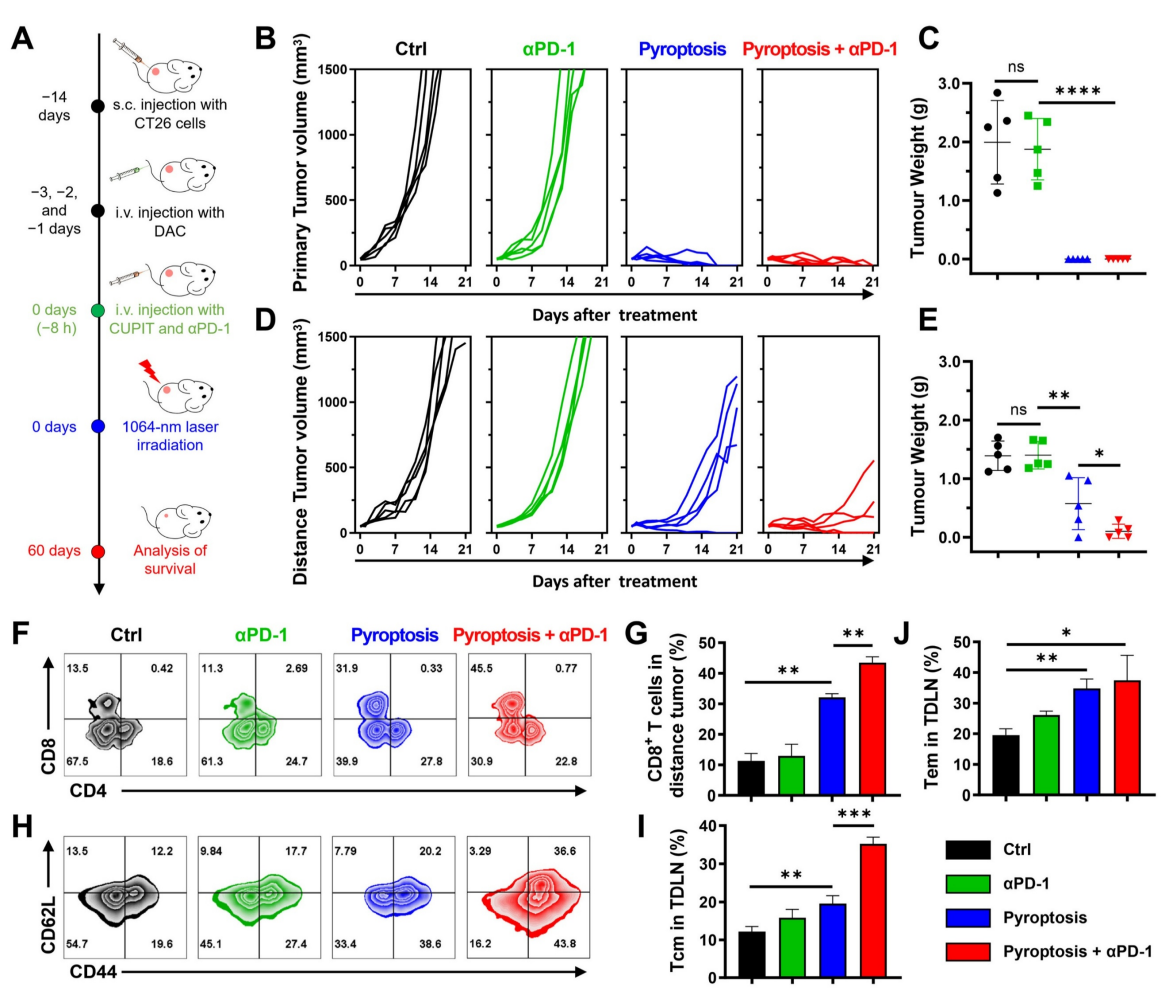
** CUPIT-mediated pyroptosis for potentiating ICB therapy.** (A) Schematic illustration of treatment schedules. (B) Tumor growth curves of primary tumor volumes after different treatments (*n* = 5). (C) Primary tumor weights at the end of experiment (*n* = 5). (D) Tumor growth curves of distance tumor volumes after different treatments (*n* = 5). (E) Distance tumor weights at the end of experiment (*n* = 5). (F) Representative flow cytometric analysis of CD8^+^ and CD4^+^ T cells gating on CD3^+^ cells in the distance tumors. (G) Representative flow cytometric analysis of memory T cells in the spleen. (H) Absolute quantification of CD8^+^ cells gating on CD3^+^ cells in distance tumor (*n* = 3). (I) Absolute quantification of central memory T cells in spleen (*n* = 3). (J) Absolute quantification of effector memory T cells in spleen (*n* = 3). Data are presented as the mean ± SD. Significance was calculated via unpaired *t*-test (ns, not significant; *P < 0.05; **P < 0.01; ***P < 0.001; ****P < 0.0001). Copper-based pyroptosis-immunotherapy trigger, CUPIT; immune checkpoint inhibitor, ICB.

Because of improved immune infiltration in mice following CUPIT-mediated pyroptotic therapy, we investigated the inhibitory effects of pyroptosis combined with immune checkpoint inhibitors (ICBs) on distant tumors. As illustrated in Figure 6A, dual-tumor mice were established to simulate primary tumor and distant metastasis. The dual-tumor mice were randomly divided into four groups including αPD-1 treatment, CUPIT-mediated pyroptotic therapy, CUPIT-mediated pyroptotic therapy plus αPD-1, and a PBS-only group as control.

The tumor growth curve demonstrated that the combination of CUPIT-mediated pyroptotic therapy and αPD-1 significantly delayed the growth of both primary and distant tumors, compared to either CUPIT or αPD-1 treatment alone. Additionally, the tumor weights at the end of the analysis period further confirmed that combination therapy effectively delayed tumor growth (Figures 6B-E). The median survival time for the PBS group was 20 days, whereas the group receiving combined CUPIT-mediated pyroptotic therapy and αPD-1 treatment exhibited a median survival time of 48 days, as shown in the survival curve (Figure S32, Supporting Information).

To further evaluate the enhancement of antitumor immunity by CUPIT-mediated pyroptosis compared to αPD-1 treatment alone, infiltrating T lymphocytes in distant tumor tissues and immunological memory cells in the spleen were systematically investigated using flow cytometry. A significant increase in the proportion of CD3^+^CD8^+^ T cells was observed in the combination therapy group compared to the αPD-1 treatment group, indicating that tumor pyroptosis effectively remodeled the TME and elicited anti-tumor immune responses (Figures 6F-G). To determine whether the immune memory effect of T cells could be enhanced by CUPIT-mediated pyroptotic therapy, central memory T cells (Tcm; CD44^+^CD62L^+^CD8^+^ T cells) and effector memory T cells (Tem; CD44^+^CD62L^-^CD8^+^ T cells) in the TDLN were measured. Significant increases in the populations of both Tcm and Tem were observed after combination therapy (Figures 6H-J). To further investigate the persistence of the antitumor immune memory induced by this combination strategy, the proportions of Tcm and Tem in the spleen were assessed at 90 days post-treatment (Figure S33, Supporting Information). Notably, sustained and significantly elevated levels of both Tcm and Tem populations were detected in the combination group, indicating robust, long-lasting immune memory. These results demonstrate that CUPIT-mediated pyroptosis not only augments acute antitumor immune responses but also supports durable immune protection. These findings suggest that the therapeutic effect of αPD-1 can be improved by CUPIT-mediated pyroptosis, providing a compelling rationale for the combination of controllable pyroptotic therapy with ICB therapy for tumor elimination.

## Conclusions

In this study, a pyroptosis proportionally tunable nanomodulator termed CUPIT was developed to induce tumor-specific and proportionally tunable pyroptosis in CRC mouse models. The PPP index was introduced to describe the relationship between real-time quantifiable PTT temperature and pyroptosis rate in tumor cells. This method enables quantification of pyroptosis *in vitro* and offers guidance for pyroptosis-mediated tumor therapy *in vivo*. Using PPP as a guide, we achieved efficient tumor killing and immune activation while minimizing systemic side effects. Our results indicate that 18.8% pyroptosis is optimal for achieving tumor elimination with minimal side effects. Moreover, CUPIT-mediated pyroptosis effectively enhanced the therapeutic effect of ICB, leading to growth inhibition of distant tumors and prolonged median survival by 1.71-fold. This study presents a novel method for controlling and quantifying pyroptosis utilizing real-time visualization and quantifiable PTT to achieve safe and effective pyroptosis-mediated antitumor therapy.

## Supplementary Material

Supplementary figures.
