# Supplementary material for: A pyroptosis proportion tunable nano-modulator for cancer immunotherapy
Source: Theranostics. 2025 Jul 25;15(16):8320–36. doi: 10.7150/thno.112209 (PMC12374590; doi:10.7150/thno.112209)
Supplement: Supplementary file 1 — Supplementary figures. [file thnov15p8320s1.pdf]

Supporting Information

A pyroptosis proportion tunable nano-modulator for cancer immunotherapy

Zhuang Chen[1,#], Zuo Yang[1,#], Zhiping Rao[1,#], Yi Luo[1], Weijing Liu[1], Chaoqiang Qiao[1], Qian Jia[1], Peng Yang[1]*, Ruili Zhang[1]*, and Zhongliang Wang[1]*

Supplementary figures

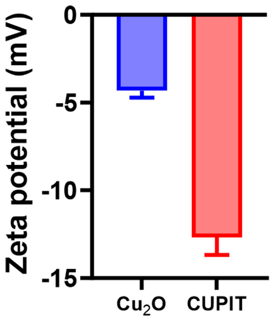

Figure S1. Hydrodynamic diameter distribution of Cu_2O and CUPIT.

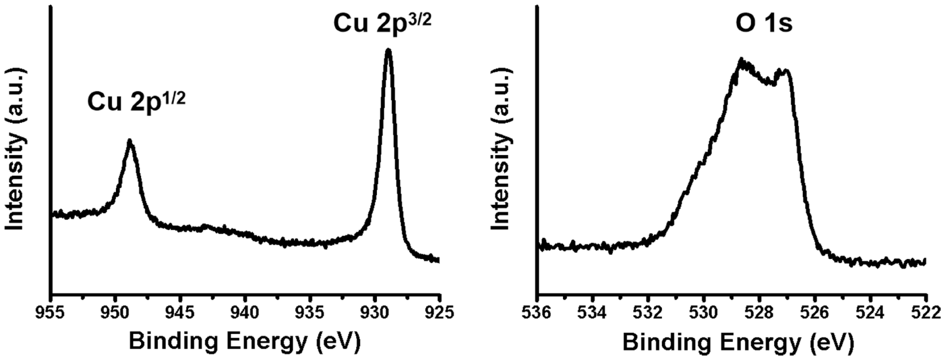

Figure S2. The XPS characterization of the CUPIT.

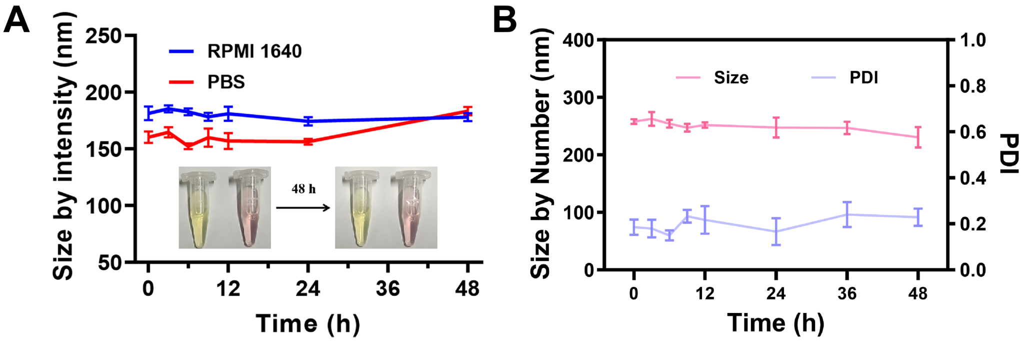

Figure S3. *In vitro* stability of CUPIT NPs in (A) PBS and RPMI 1640 within 48h; (B) Size and PDI change of CUPIT in 10% FBS within 48h (n=3).

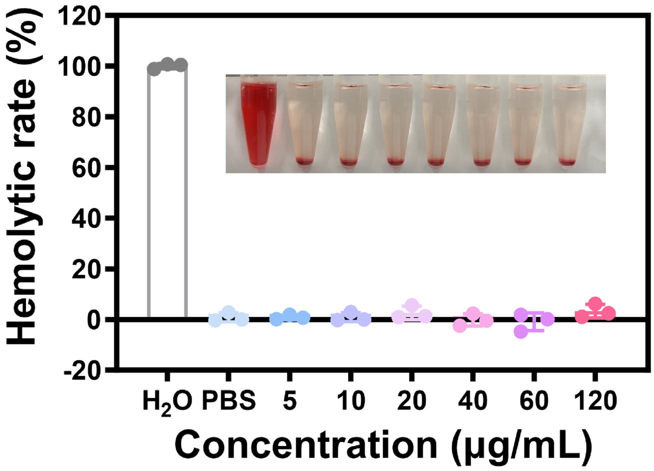

Figure S4. Hemolysis rates of nanoparticles at different concentrations, Positive control: red blood cells treated with deionized water. Negative control: red blood cells treated with PBS. (n=3).

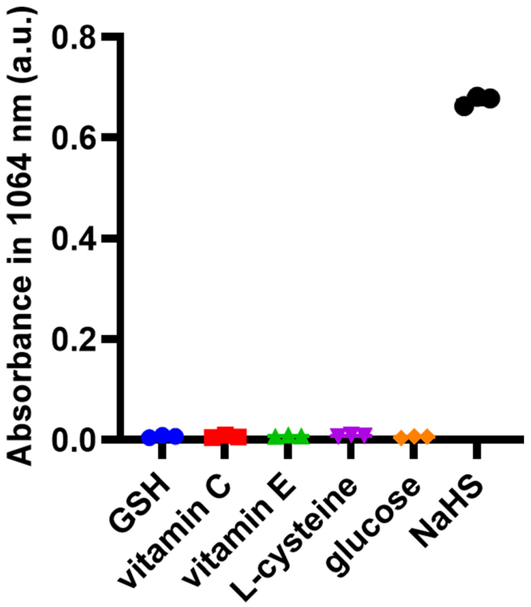

Figure S5. Response specificity of the CUPIT with the reducing substances in body.

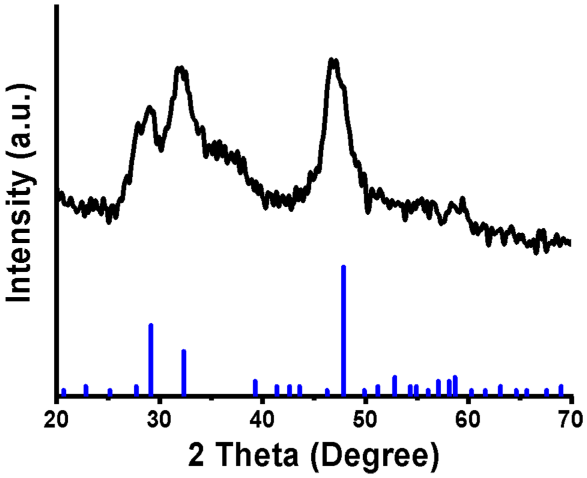

Figure S6. XRD characterization of CUPIT in response to NaHS.

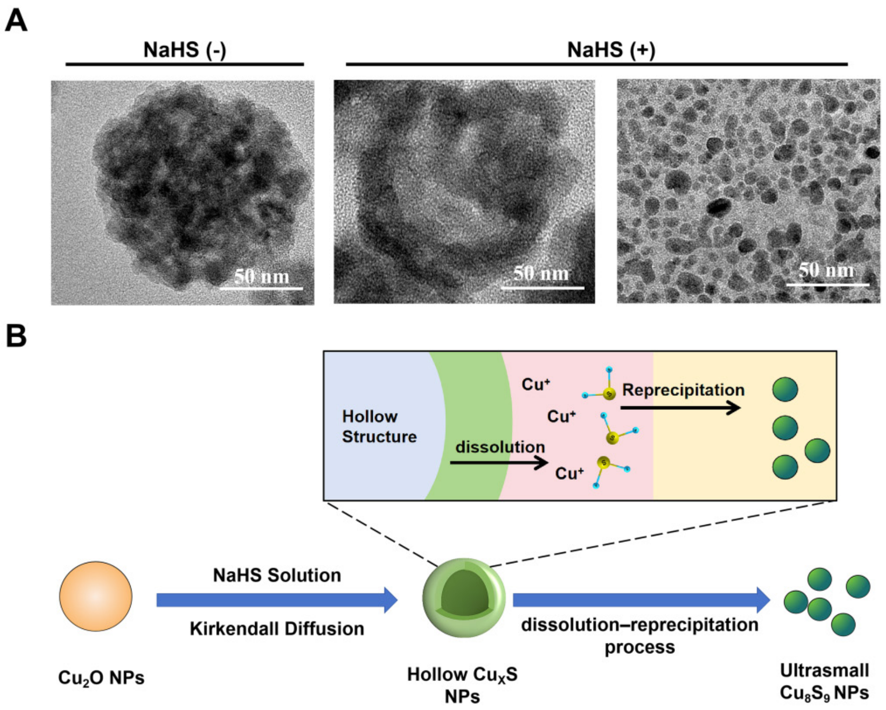

Figure S7. (A) TEM images of CUPIT NPs before and after reaction with NaHS; (B) Proposed mechanism of Cu_2O sulfidation to form Cu_8S_9.

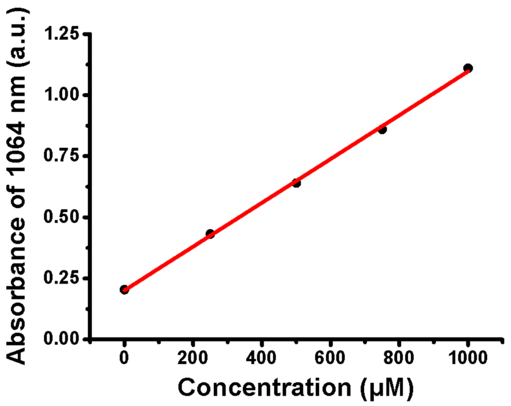

Figure S8. The absorbance of CUPIT NPs at 1064 nm in response to different concentrations of NaHS (0-1000 μM).

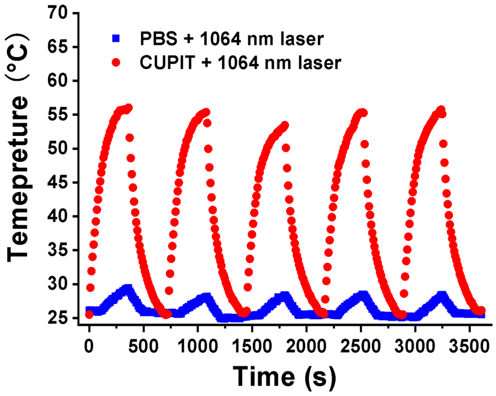

Figure S9. The photothermal cycle curve of the CUPIT NPs and PBS.

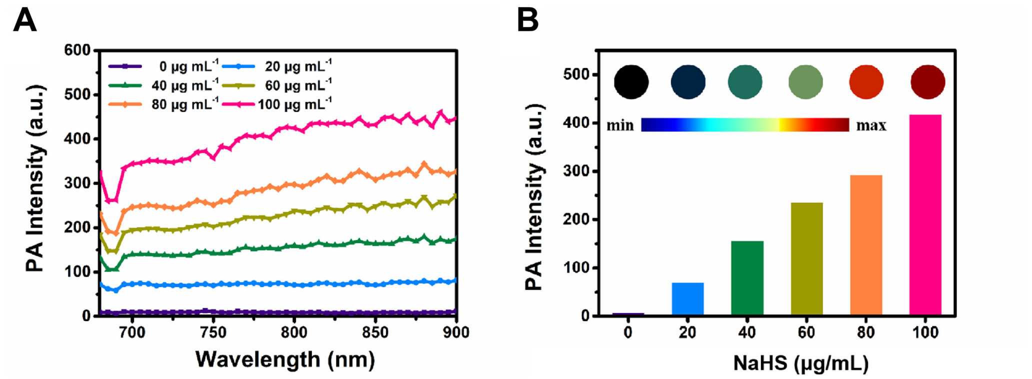

Figure S10. *In vitro* photoacoustic performance characterization of CUPIT NPs in the presence of NaHS. (A) photoacoustic signal spectrum after reaction of CUPIT with different concentrations of NaHS; (B) photoacoustic signal intensity at 800 nm after reaction of CUPIT with different concentrations of NaHS, the legend shows the reconstructed signal image.

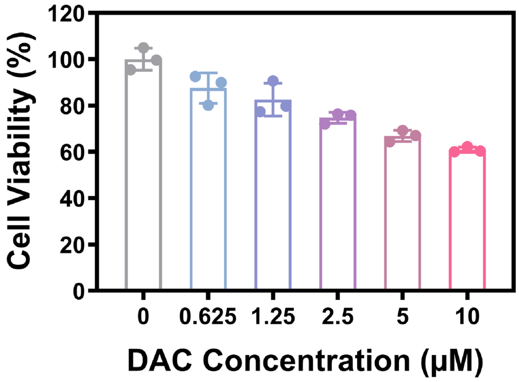

Figure S11. Cell viability of CT26.WT cells after incubation with different concentrations of DAC (n=3).

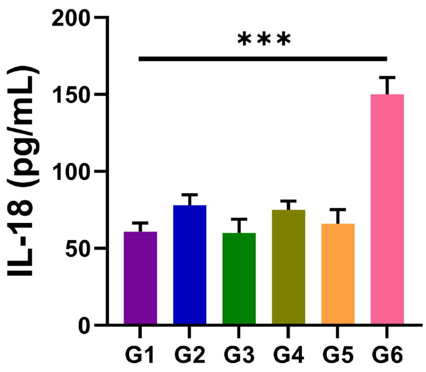

Figure S12. The release of IL-18 after different treatments (n = 3). Data are presented as the mean ± SD. Significance was calculated via unpaired t-test (***P < 0.001).

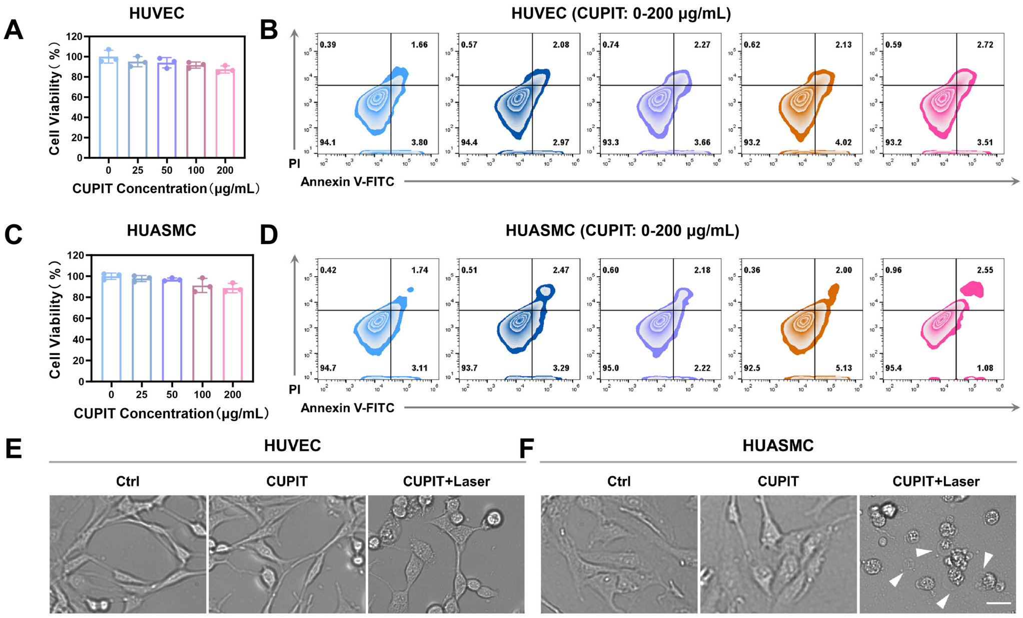

Figure S13. (A) Cell viability assay of HUVEC cells (low GSDME expression) after incubation with increasing concentrations of CUPIT (n=3). (B) Flow cytometry analysis of HUVEC cells stained with PI and Annexin V-FITC following treatment with various concentrations of CUPIT. (C) Cell viability assay of HUASMC cells (high GSDME expression) after incubation with increasing concentrations of CUPIT (n=3). (D) Flow cytometry analysis of HUASMC cells stained with PI and Annexin V-FITC after CUPIT treatment at different concentrations. (E) Representative images of HUVEC cells after CUPIT-mediated photothermal therapy. (F) Representative images of HUASMC cells after CUPIT-mediated photothermal therapy, scale bar: 25 μm.

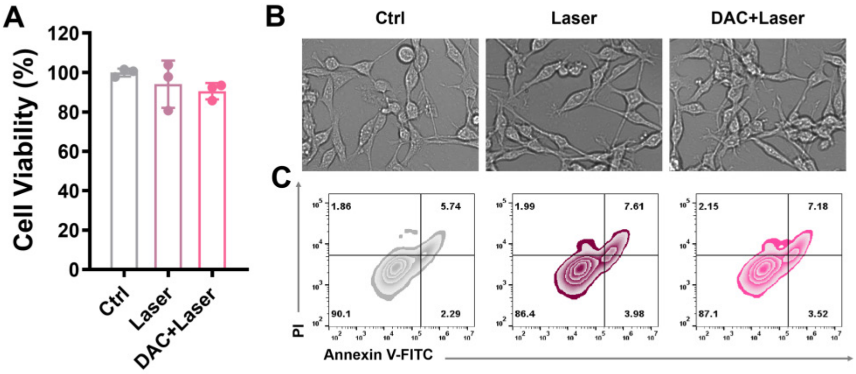

Figure S14. (A) Cell viability of CT26.WT cells after laser irradiation (n=3). (B) Representative images showing the morphological changes of CT26.WT cells after laser irradiation. (C) Flow cytometry analysis of CT26.WT cells stained with PI and Annexin V-FITC following laser irradiation.

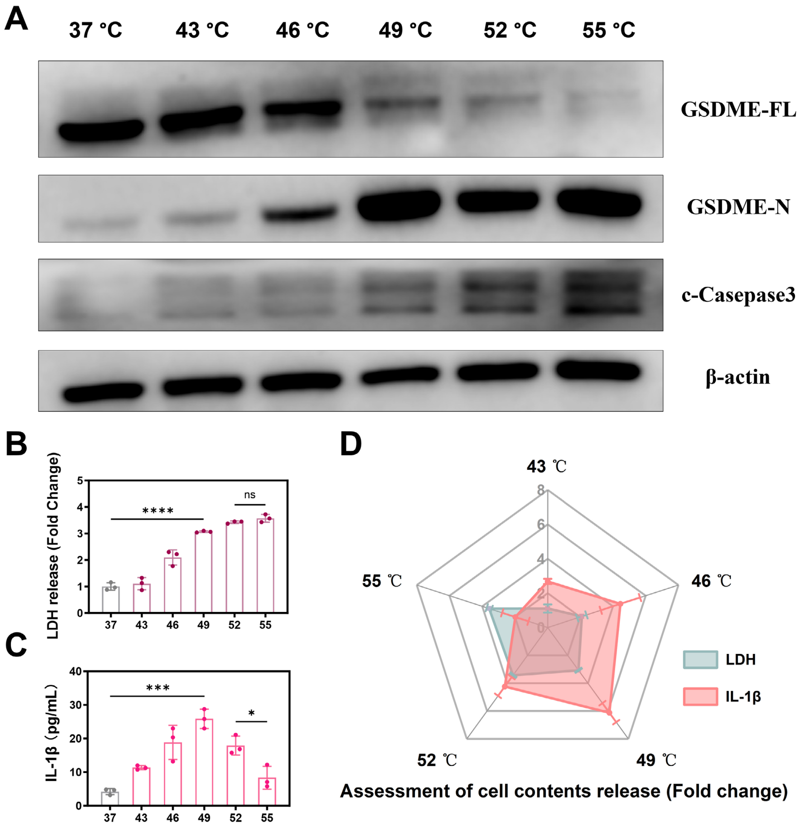

Figure S15. (A) Western blot analysis of GSDME-FL, GSDME-N, and c-Caspase-3 in CT26.WT cells after photothermal treatment at various temperatures. (B) LDH release from CT26.WT cells following photothermal therapy at different temperatures (n=3). (C) IL-1β release from CT26.WT cells following photothermal therapy at different temperatures (n=3). (D) Visual representation of LDH and IL-1β release from CT26.WT cells under different photothermal conditions (n=3). Data are presented as the mean ± SD. Significance was calculated via unpaired *t*-test (****P* < 0.001, *****P* < 0.0001).

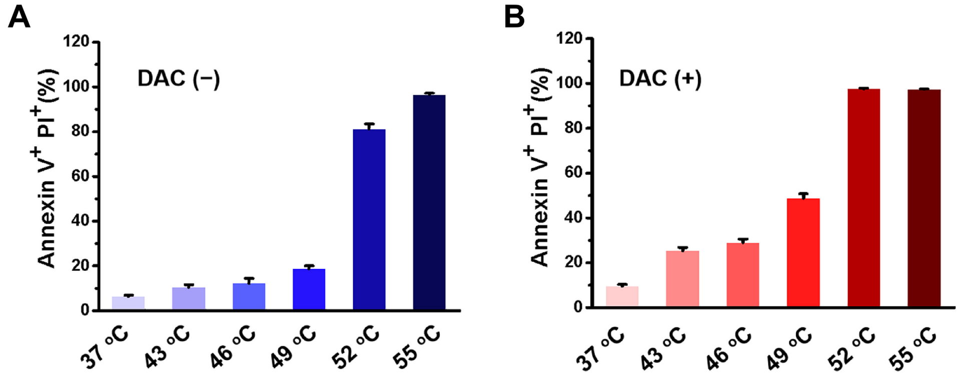

Figure S16. Proportion of CT26.WT cell death at different temperatures. (A) without DAC pretreatment; (B) with DAC pretreatment.

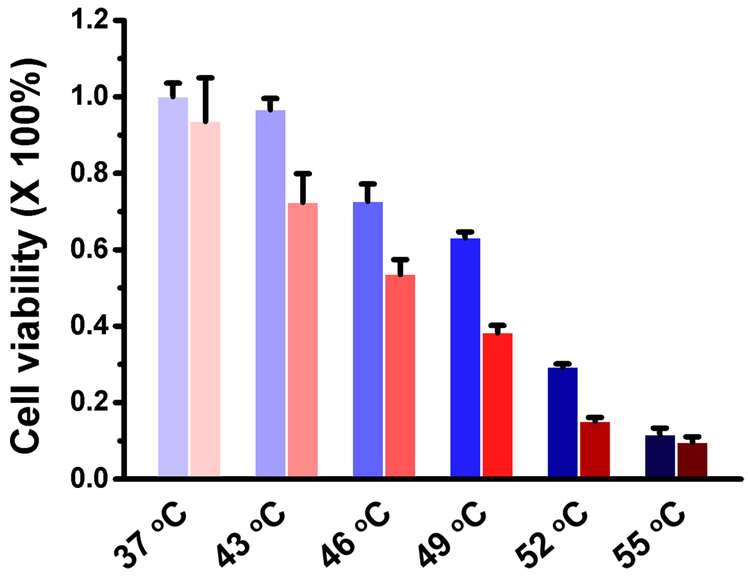

Figure S17. The viability of CT26.WT cells at different temperatures (blue: with DAC pretreatment; red: without DAC pretreatment).

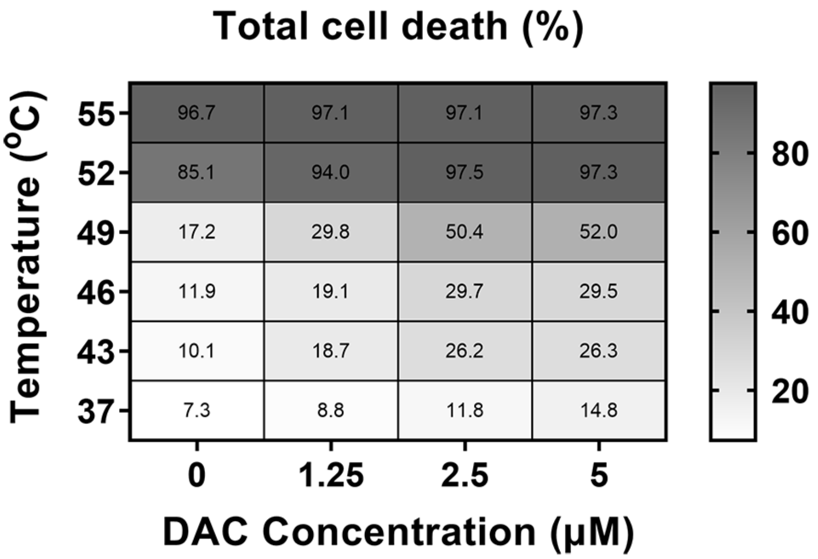

Figure S18. Heat map of TCD induced by different temperatures under pretreatment with different concentrations of DAC.

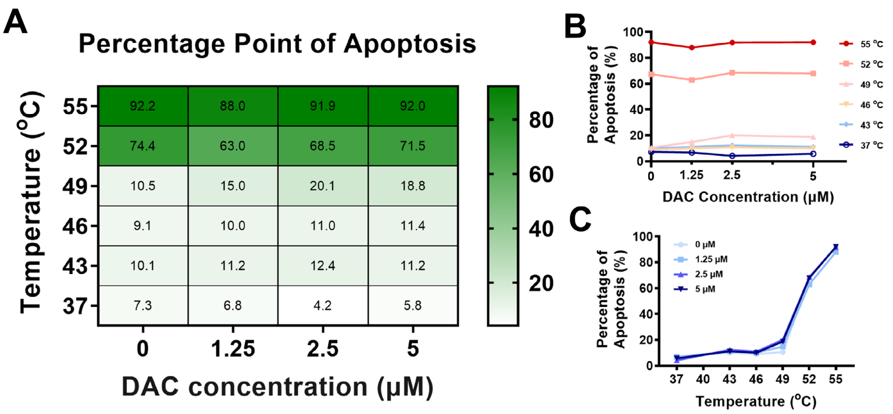

Figure S19. (A) Heat map of PPA induced by different temperatures under pretreatment with different concentrations of DAC; (B) PPA after pretreatment with different concentrations of DAC; (C) PPA treated with different temperatures.

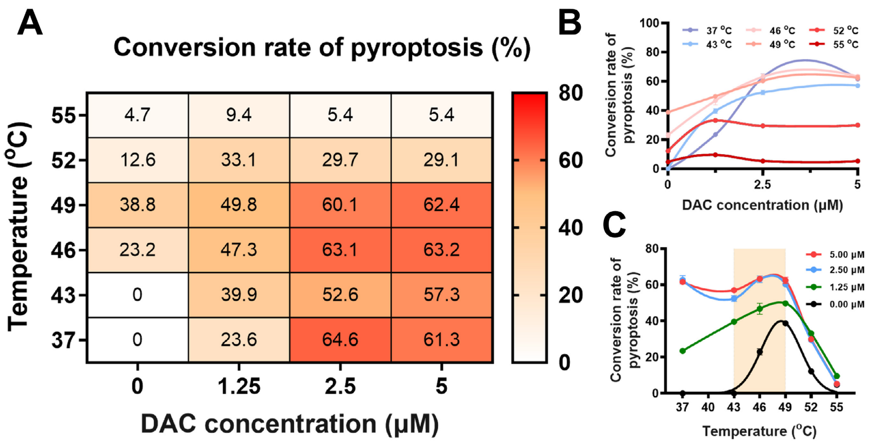

Figure S20. (A) Heat map of CRP induced by different temperatures under pretreatment with different concentrations of DAC; (B) CRP after pretreatment with different concentrations of DAC; (C) CRP treated with different temperatures.

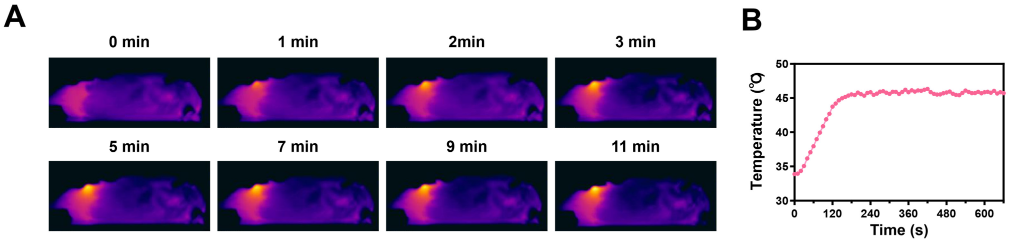

Figure S21. (A) Infrared thermal images of CT26.WT tumor-bearing mice under photothermal treatment. (B) Photothermal heating curve showing the temperature of the tumor region maintained at 46 °C during irradiation.

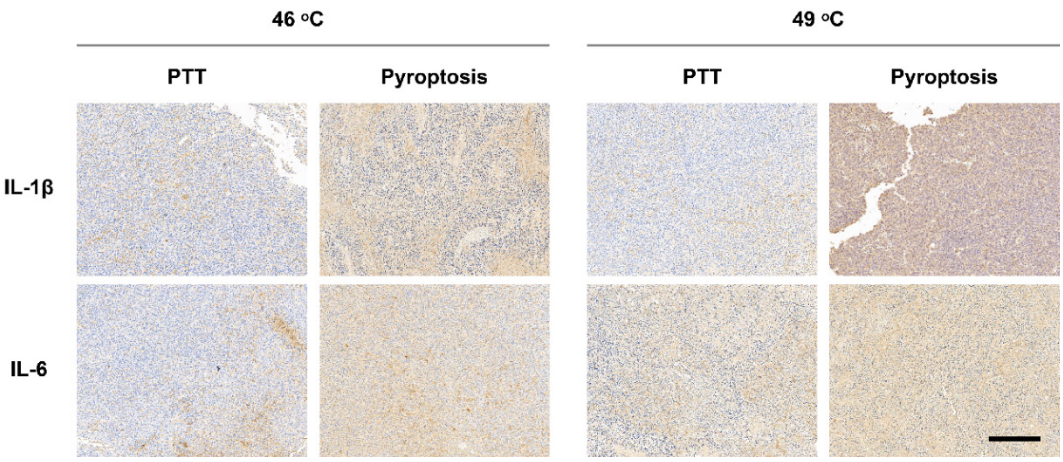

Figure S22. Immunohistochemical analysis of IL-1β and IL-6 expression in tumor tissues following photothermal therapy (CUPIT alone) and pyroptosis treatment (CUPIT combined with DAC pre-treatment) at 46 °C and 49 °C, scale bar: 200 μm.

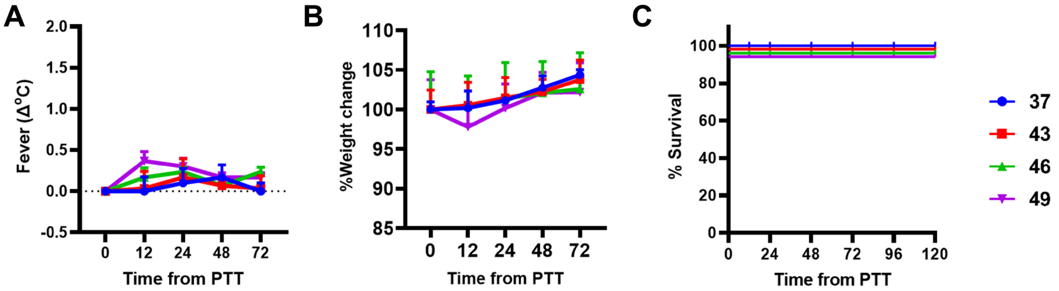

Figure S23. (A) Body temperature changes and (B) weight loss of mice after different temperatures treatment (n = 5). (C) survival curves of the mice after different temperatures treatment (n = 5).

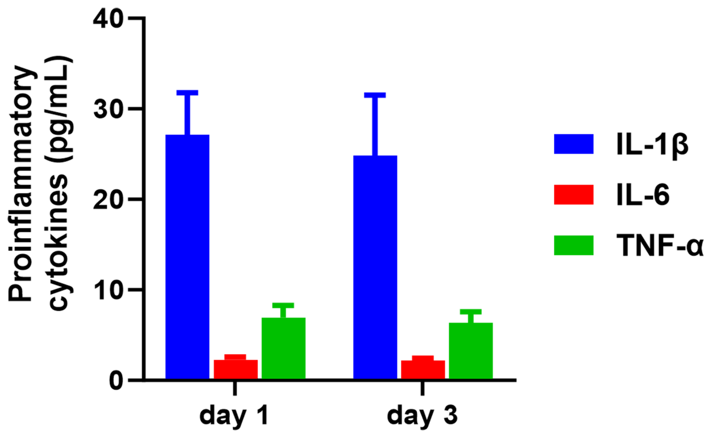

Figure S24. Changes in serum IL-1β, IL-6 and TNF- α levels induced under 49 °C conditions in non-DAC pretreated mice.

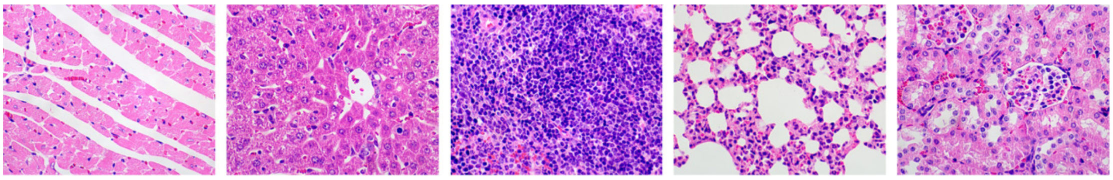

Figure S25. Hematoxylin and eosin stain of mouse organs after photothermal treatment at different temperatures (from left to right: heart, liver, spleen, lung and kidney).

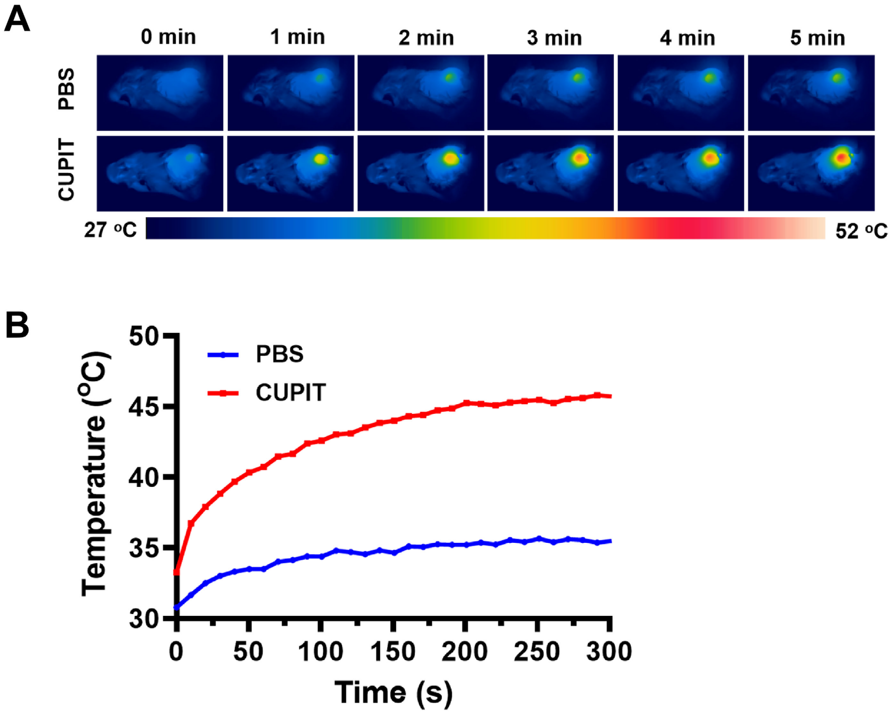

Figure S26. a) Photothermal imaging of CUPIT in the CT26.WT tumor-bearing mouse model; b) characterization of the photothermal heating curve of CUPIT in the CT26.WT tumor-bearing mouse model.

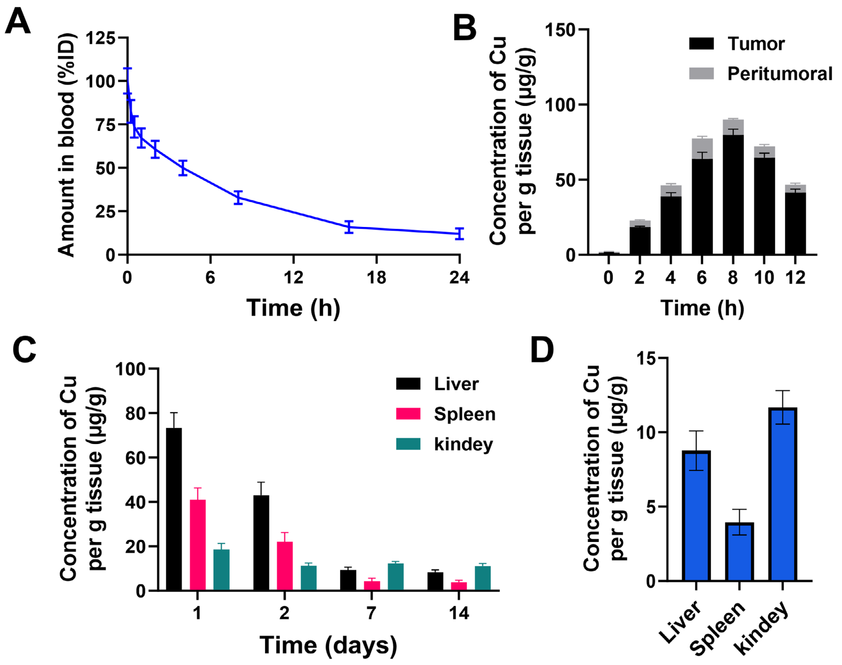

Figure S27. (A) Pharmacokinetic profile of CUPIT following intravenous administration, displaying plasma concentration over time (n=3). (B) Tumor accumulation of CUPIT at various time points, illustrating its tumor-targeting capability (n=3). (C) Biodistribution and metabolism of CUPIT in the liver, spleen, and kidney at different time points after injection (n=3). (D) Baseline copper content in normal liver, spleen, and kidney tissues (n=3). Data are presented as the mean ± SD.

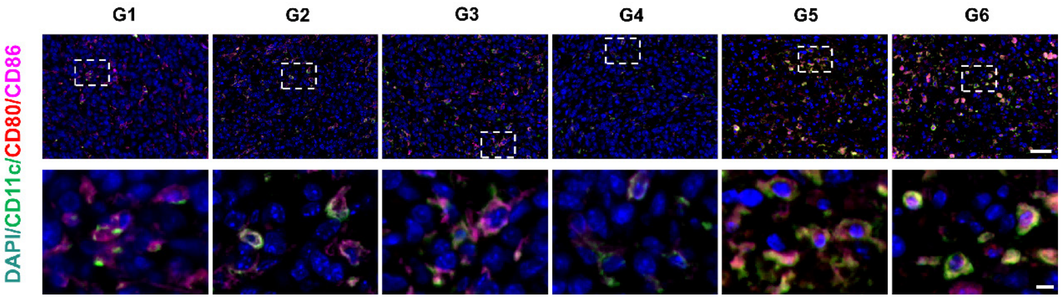

Figure S28. Infiltration of mature dendritic cells in tumor tissues after different treatments as assessed by immunofluorescence, scale bar: 200 μm and 50 μm.

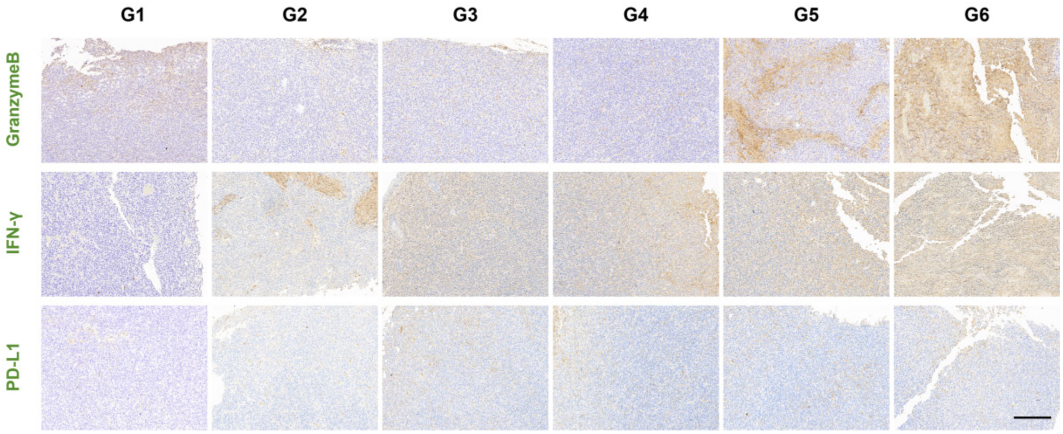

Figure S29. Immunohistochemical analysis of PD-L1, IFN-γ, and Granzyme B expression in tumor tissues from different treatment groups at the end of therapy, scale bar: 200 μm.

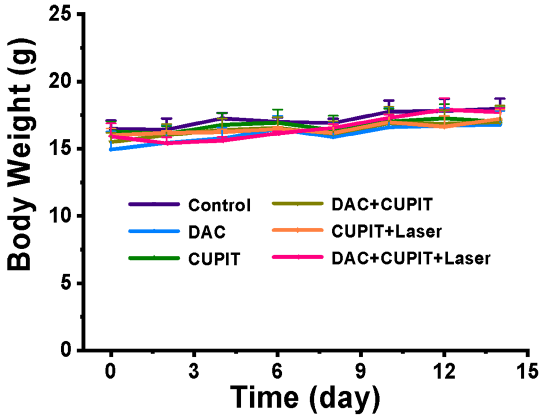

Figure S30. Body weight changes of mouse in different group within 14 days.

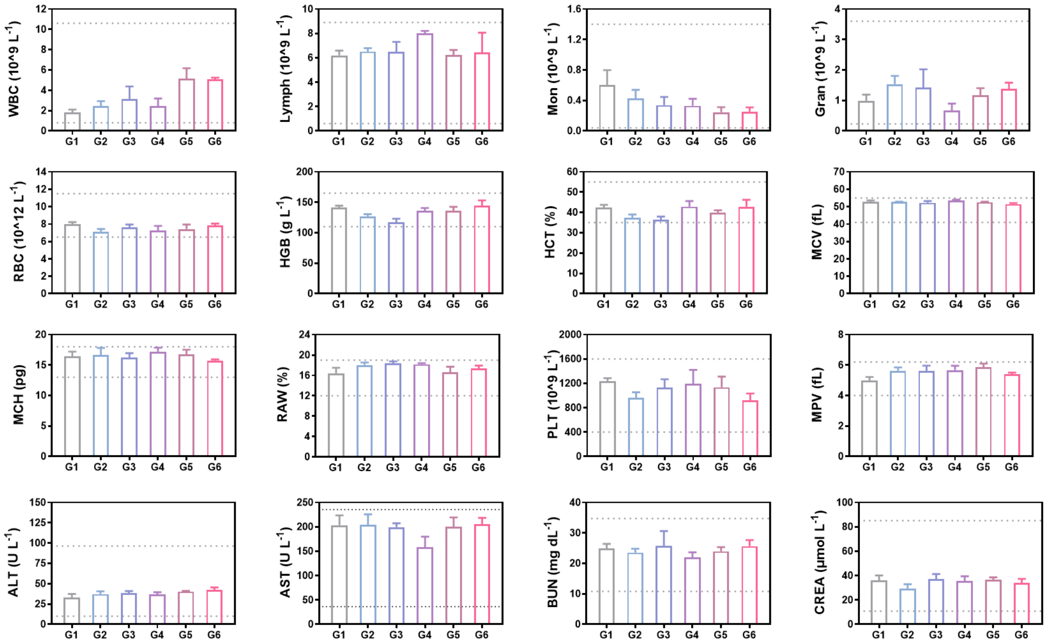

Figure S31. Hematological and serum biochemical parameters of mice from different treatment groups at the end of therapy (n=3). Data are presented as the mean ± SD.

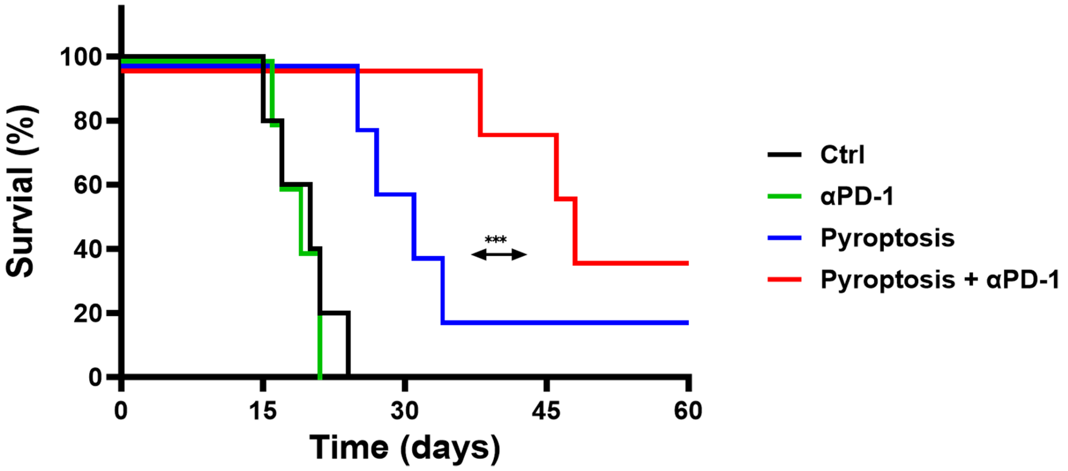

Figure S32. Survival rate of mice receiving different treatments (n = 5).

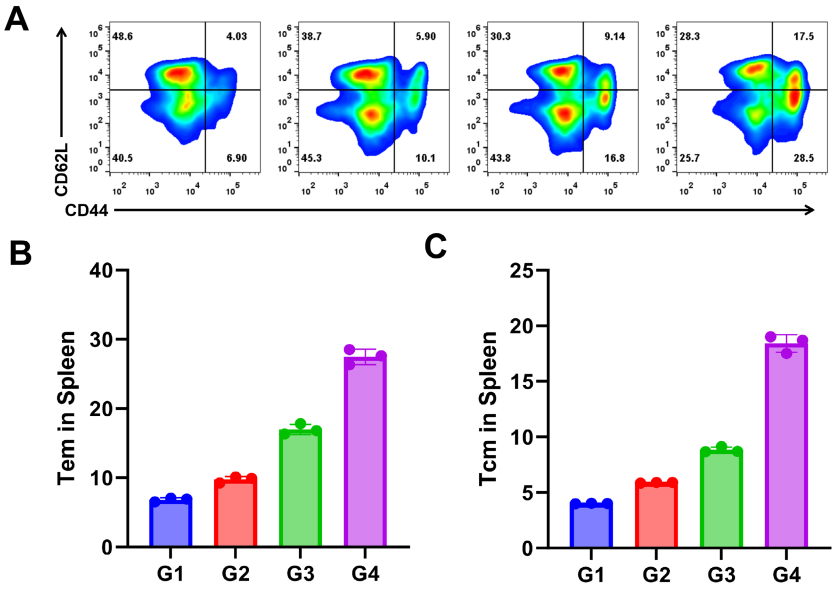

Figure S33. (A) Tumors were surgically removed 5 days after completion of treatment, and spleens were collected and analyzed by flow cytometry on day 90 to assess memory T cell populations. (B) Proportion of effector memory T cells (Tem) in spleens of mice from each treatment group as determined by flow cytometry (n=3). (C) Proportion of central memory T cells (Tcm) in spleens of mice from each treatment group as determined by flow cytometry (n=3). Data are presented as the mean ± SD.
